# TBL1XR1 Ensures Balanced Neural Development Through NCOR Complex-Mediated Regulation of the MAPK Pathway

**DOI:** 10.3389/fcell.2021.641410

**Published:** 2021-02-23

**Authors:** Giuseppina Mastrototaro, Mattia Zaghi, Luca Massimino, Matteo Moneta, Neda Mohammadi, Federica Banfi, Edoardo Bellini, Marzia Indrigo, Giulia Fagnocchi, Anna Bagliani, Stefano Taverna, Maria Rohm, Stephan Herzig, Alessandro Sessa

**Affiliations:** ^1^Stem Cell and Neurogenesis Unit, Division of Neuroscience, IRCCS San Raffaele Scientific Institute, Milan, Italy; ^2^Neurimmunology Unit, Division of Neuroscience, IRCCS San Raffaele Scientific Institute, Milan, Italy; ^3^Medical Oncology Unit, ASST Ovest Milanese, Legnano Hospital, Legnano, Italy; ^4^Institute for Diabetes and Cancer IDC, Helmholtz Center, Munich, Germany; ^5^Joint Heidelberg-IDC Translational Diabetes Program, Inner Medicine 1, Heidelberg University Hospital, Heidelberg, Germany; ^6^Medical Faculty, Technical University Munich, Munich, Germany; ^7^German Center for Diabetes Research, Oberschleissheim, Germany

**Keywords:** TBL1XR1, NCOR, brain development, neurodevelopmental disorders, MAPK

## Abstract

*TBL1XR1* gene is associated with multiple developmental disorders presenting several neurological aspects. The relative protein is involved in the modulation of important cellular pathways and master regulators of transcriptional output, including nuclear receptor repressors, Wnt signaling, and MECP2 protein. However, TBL1XR1 mutations (including complete loss of its functions) have not been experimentally studied in a neurological context, leaving a knowledge gap in the mechanisms at the basis of the diseases. Here, we show that *Tbl1xr1* knock-out mice exhibit behavioral and neuronal abnormalities. Either the absence of TBL1XR1 or its point mutations interfering with stability/regulation of NCOR complex induced decreased proliferation and increased differentiation in neural progenitors. We suggest that this developmental unbalance is due to a failure in the regulation of the MAPK cascade. Taken together, our results broaden the molecular and functional aftermath of TBL1XR1 deficiency associated with human disorders.

## Introduction

The building of a healthy nervous system is due to a concerted array of molecular mechanisms within and between different cell types during the embryonic life and beyond (Borrell, 2019). Not surprisingly, a huge variety of gene mutations is associated with neurodevelopmental disorders (Thapar et al., 2017). *TBL1XR1*, encoding for transducin β-like 1—related protein 1 (a member of HDAC containing NCOR/SMRT complexes) (Yoon et al., 2003), has been associated with different human developmental diseases, spanning from autism spectrum disorders (ASD) (O’Roak et al., 2012; Stessman et al., 2017), to West syndrome (Saitsu et al., 2014), schizophrenia (SCZ) (Nishi et al., 2017), intellectual disability (Pons et al., 2015; Riehmer et al., 2017). Mutations leading to the substitution Y446C of *TBL1XR1* are the only genetic cause of Pierpont syndrome (Heinen et al., 2016). The *TBL1XR1* mutational spectrum is wide, ranging from deletions to duplications and point mutations, all inducing frameshifts or amino-acidic substitutions (Li et al., 2015). However, to our knowledge the hypothesis that mutations in *TBL1XR1* gene directly lead to neurological impairment—and whether this is due to the disruption of the function of NCOR complex—has not been investigated in high systems (e.g., mammals).

Co-repressors NCOR1 and NCOR2 (in mice NCOR and SMRT, respectively), contribute to different repressive pathways, including the inhibition of the downstream cascade of nuclear receptors such as the receptors of thyroid hormone (TRs) and retinoic acid (RARs), in the absence of their ligands (Kong et al., 2020). While the two proteins are very similar (sharing portions of sequence and domains), they play slightly different roles in different cell populations (Iemolo et al., 2020; Sun and Xu, 2020). For example, during brain development both factors are required for the proliferation capability of neural precursors, with NCOR1 loss specifically impacting on premature differentiation of astrocytes and oligondendroglia (Castelo-Branco et al., 2014), while the absence of NCOR2 leads to early differentiation to neurons and astrocytes (Hermanson et al., 2002; Jepsen et al., 2007). NCOR complexes are believed to mediate repression through the deacetylation of specific chromatin regions through a direct action of HDAC proteins, particularly HDAC3 (Zhuang et al., 2018). In this context, TBL1XR1 and its related member TBL1X—both WD40 repeat containing proteins—are important for both formation and dismantling of the NCOR complex. Indeed, they are required both for the repressive activity of the complex and the ubiquitination-dependent dismissal of the co-repressors in response to appropriate signals (e.g., the ligands of a nuclear receptor), or even when the co-repressors are missing (Perissi et al., 2004; Ishizuka and Lazar, 2005; Yoon et al., 2005; Li et al., 2015). Importantly, TBL1XR1 and TBL1X play either redundant or exclusive roles in these processes, depending on the contexts and/or the targets (Perissi et al., 2008). Both TBL1XR1, TBL1X, and the NCOR complexes are physically associated with the Rett syndrome’s causative factor MECP2, further underlying the importance of these molecules for normal brain functioning (Ebert et al., 2013; Lyst et al., 2013; Kruusvee et al., 2017). Despite TBL1XR1 and the related complexes have been known for years (Perissi et al., 2010), their specific role in brain development and possible causality in the neurological aspects of human diseases have been only marginally explored.

In the present study, we describe how of the lack of TBL1XR1 affects mouse behavior, brain development and function. We show that TBL1XR1 loss of function impacts on coordination, memory, and sociability, similarly to what occurs in humans. Mutant neural progenitors proliferated less than normal, due to the lack of NCOR-mediated regulation of MAPK cascade. The reductionist loss-of-function approach was enriched by the usage of disease-specific mutant proteins for complementation experiments. This analysis indicated how point mutations are generally different from the *Tbl1xr1* knock-out (KO) condition, except for the F10L mutation that was retrieved in SCZ cases. Altogether, our data indicate a broad impact of TBL1XR1 for correct neuronal development and maturation, accounting for the complex and variegated spectrum of pathological traits associated with the different *TBL1XR1* mutations in humans.

## Materials and Methods

### Mice

*Tbl1xr1* floxed animals (Rohm et al., 2013) and CMV:Cre were maintained by crossing each other or with backcrossing with C57BL/6 animals at the San Raffaele Scientific Institute Institutional mouse facility. Experiments were performed in accordance with experimental protocols approved by local Institutional Animal Care and Use Committee (IACUC). Experimental subjects were sacrificed by means cervical dislocation. Both total body and brains were weighted to calculate their ratio.

### Western Blot Analysis

Brain tissue and *in vitro* cells were prepared as previously indicated (Sessa et al., 2019) for western blot analysis. The following primary antibodies were used: anti-TBL1XR1 (Novus Biological NB600-270); anti-NCOR1 (Merck-Millipore ABE251); anti-HDAC3 (Abcam ab 13704); anti-GAPDH (Abcam ab8245); anti-βCATENIN (Chemicon AB19022); anti-pβCATENIN (Ser33/37/Thr41, Cell signaling #9561S); anti-ERK1 (Cell Signaling Technology 4372); anti-pERK1 (Cell Signaling Technology 5726); Anti-V5 (Thermo Fisher Scientific R960-25); Anti-Histone H3 (Abcam, ab1791).

### Behavioral Testing

Animals were housed at a constant temperature of 23°C in a 12 h light/dark cycle (lights off at 7 PM), with food and water available *ad libitum*. We analyzed control and mutant littermates (males only) ranging from 2 to 4 months of age. The operator was blind to the genotype.

#### Rotarod

Mice were assessed for the latency to fall as previously described (Sessa et al., 2019).

#### Beam Walking

Beam crossings and number of paw slips were assessed as previously described (Sessa et al., 2019).

#### Open Field

Mice were located in a square arena (50 × 50 cm) and video-recorded for 10 min. Total distance traveled and the time spent near the walls were scored by EthoVisionXT software (Noldus Information Technology, Wageningen, Netherlands).

#### Catwalk

Motor function was estimated through the CatWalk system (Noldus Information Technology, Wageningen, Netherlands). The animal paw prints were recorded as the animal moved across a walkway with an illuminated glass floor fitted with high-speed video camera assembled with 8.5 mm wide-angle lenses below the floor. The day before the test, each animal was placed on the CatWalk platform to walk freely as habituation. During the test, three uninterrupted runs (minimum of 5-step sequence patterns) were collected. Several gait parameters were calculated and analyzed by the dedicated software using the position, pressure and surface area of each paw footprint.

### Marble Burying Test

Individual animals were placed in a cage (20 × 32cm) with 5 cm of bedding material and 12 marbles (12–15 mm in diameter). The marbles were placed in a 3 × 4 matrix. The number of buried marbles was counted after a 30 min session.

### 8-Arm Radial Maze

**FIGURE 1:**
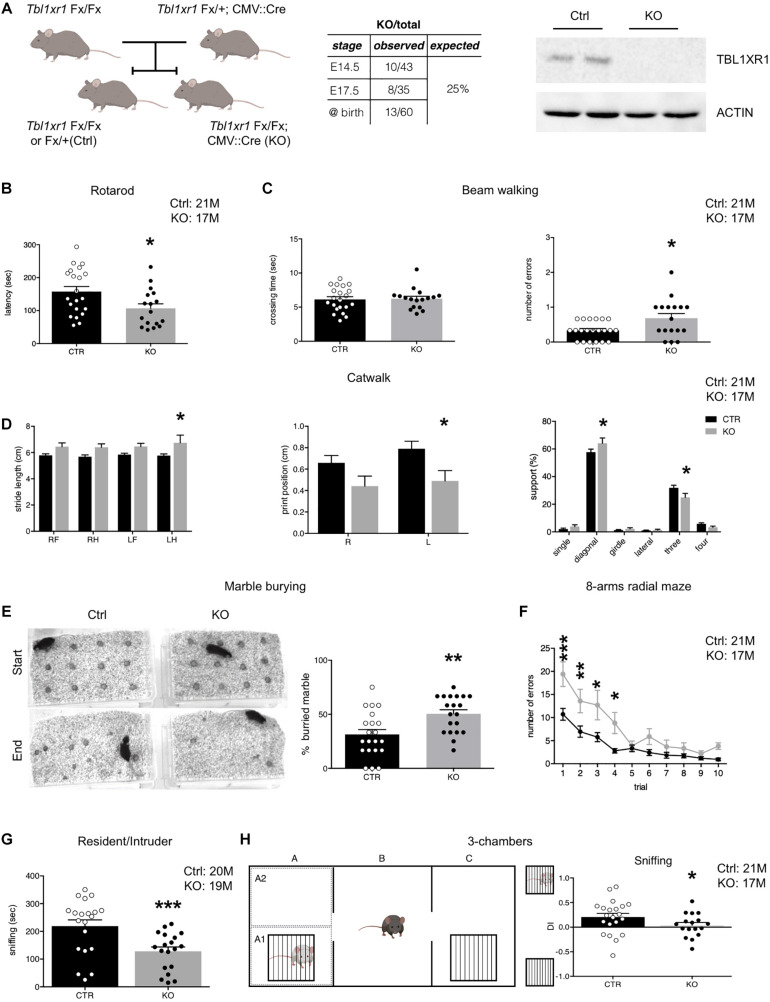
Behavioral deficit of Tbl1xr1 mutant mouse. **(A)** Breeding strategy for obtaining both *Tbl1xr1* mutant and control (left), table summarizing mutant animals found at the indicated developmental stages (center) and Western blot for TBL1XR1 protein in control and mutant brain tissue (right). **(B)** Rotarod motor test (shown as mean + s.e.m. with dots representing individual samples): *n* (adult male mice): Ctrl = 21, KO = 17, *p = 0.02, statistically compared using unpaired *t-*test. **(C)** Beam walking assay in which time of crossing and number of errors (paw falls) have been quantified (shown as mean + s.e.m. with dots representing individual samples): crossing time (top): *n* (adult male mice): Ctrl = 21, KO = 17, *p* = 0.9933, statistically compared using Mann-Whitney; errors (bottom): n (adult male mice): Ctrl = 21, KO = 17, *p = 0.0126, statistically compared using unpaired *t*-test. **(D)** Through catwalk assay we measured stride length, position of the paw during walking (print position) and support that the animal has during walking (shown as mean + s.e.m.), n (adult male mice): Ctrl = 21, KO = 17: *p < 0.05. Statistically compared using 2-way ANOVA. **(E)** Marble burying test in which the percentage of buried marble spheres has been quantified (shown as mean + s.e.m. with dots representing individual samples), n (adult male mice): Ctrl = 21, KO = 20: **p = 0.0028, statistically compared using unpaired *t*-test. **(F)** Eight-arm radial maze test quantified in which the percentage of errors (entries in arms already visited) on total visits has been quantified (shown as means ± SEMs in each experimental day, 1 trial/day), *n* (adult male mice): Ctrl = 21, KO = 17: Multiple comparisons: day 1: ***p = 0.0001; day 2: **p = 0.007; day 3: **p = 0.0039: day 4: *p = 0.021; day 5: *p* > 0.9999; day 6: *p* = 0.7603; day 7: *p* > 0.9999; day 8: *p* > 0.9999; day 9: *p* > 0.9999; day 10: *p* > 0.9999; statistically compared using 2-way ANOVA and Bonferroni’s *post hoc* test. **(G)** Sociability of adult animals measured as the time spent to interact with a novel mouse in the resident/intruder test (shown at right as means ± SEMs, with dots representing individual samples), *n* (adult male mice): Ctrl = 20, KO = 19: ***p = 0.0009; statistically compared using unpaired *t*-test. **(H)** Three-chamber test (scheme at left) quantified as percentage of the time spent sniffing the mouse enclosed in the little cage vs. the empty cage shown as discrimination index (DI) (shown as means ± SEMs, with dots representing individual samples), *n* (adult male mice): Ctrl = 21, KO = 17: sniffing: *p = 0.0287, statistically compared using unpaired *t*-test. See also Supplementary Figure S1.

8-arm radial maze tests were conducted as previously described (Sessa et al., 2019). Days 4–13 are shown as experimental days 1–10 in Figure 1F and Supplementary Figure S1D. Working memory errors (Supplementary Figure S1D) were calculated as re-entries to arms where the pellet had already been consumed.

### Elevated Plus Maze

Elevated plus maze was performed as described (Leo et al., 2014). Briefly, mice were placed in a plus-shaped maze composed by two enclosed and two open arms. The apparatus was elevated from the ground. The animals were allowed to freely explore the environment for 5 min and the time they spent in the open arms was calculated as an inverse measure of the levels of anxiety.

### Social Interaction (Resident-Intruder Test)

To elicit social interactions, one mouse (experimental subject, resident) of a pair was placed in a neutral cage and allowed to freely explore for 10 min. At the end of the session, a second male mouse (intruder) of different strain was added to the cage and the behavior of the pair of animals was recorded for 20 min. The amount of time during which the resident mice engaged in social interactions (e.g., sniffing, following, grooming, biting, chasing, mounting, wrestling) was recorded on videotapes for each pair of mice. Since the test was originally designed for testing aggressiveness, we also evaluated the time that the animals spent in aggressive behavior.

### 3-Chamber Test

Adult mice were tested using a 3-chamber test coupled with the video tracking software Ethovision XT (Noldus) as previously described (Sessa et al., 2019).

Sniffing preference was assesses using a discrimination index (DI), i.e., the difference between the sniffing time in the occupied cage and the empty one, divided by total sniffing time.

### Histology

Histological procedures to measure morphological parameters were conducted as previously described (Sessa et al., 2019).

### Immunohistochemistry

Immunohistochemical analyses were performed as previously described (Colombo et al., 2006). The primary antibodies utilized were the following: anti-CTIP2 (Abcam ab18465), anti-CUX1 (santa Cruz Biotechnology sc-13024), anti-SATB2 (Abcam, ab51502), anti-PAX6 (Covance #PRB-278P), anti GFAP (chicken, 1:1,000, Abcam, ab4674), anti-DCX (rabbit, 1:1,000, Abcam, ab18723), antiKI67 (rabbit, 1:500, immunological sciences, mab-90948), anti-NEUN (rabbit, 1:500, Abcam, ab104225).

Secondary antibodies: 488-mouse (donkey, 1:2,000, Molecular Probes, A21202), 488-rabbit (donkey, 1:2,000, Molecular Probes, A21026), 594-mouse (donkey, 1:2,000, Molecular Probes, A10036), 594-rabbit (donkey, 1:2,000, Molecular Probes, A21207). DAPI (4′,6′-diamidino-2-phenylindole) was used as nuclear counterstaining.

### Electrophysiology

All procedures were approved by the Italian Ministry of Health and the San Raffaele Scientific Institute Animal Care and Use Committee in accordance with the relevant guidelines and regulations. We analyzed mice of both sexes (30 days of age) as previously described (Sessa et al., 2019).

### Primary Neuronal Cultures

Primary cultures of mouse embryonic hippocampal neurons were prepared from E17.5 mutant and control mice as previously described (Sessa et al., 2019).

### Sholl Analysis

Neuronal cultures were transduced with lentiviral vector EF1a-GFP at a low titer the day after the plating for 1 h, in order to obtain sparse GFP cell-labeling. At the desired time points, cells were processed for IF analysis. Images of the dendritic tree of double positive GFP+/MAP2+ neurons were investigated using Sholl Analysis plug-in (Ferreira et al., 2014) in Fiji software (NIH, United States).

### *In vitro* Spine Analysis

Mutant and control primary cultures (E17.5 murine hippocampal neurons) were infected with lentiviral vector EF1a-GFP at a low titer at DIV1 for 1 h to get few and sparse GFP labeled cells. At DIV 15 and DIV 21, the primary neurons were stained with GFP and analyzed at confocal microscope. Spine density was measured in both mutant and wild type neurons.

### Golgi-Cox Staining and Spine Measurements

Golgi-Cox staining was performed as previously described (Sessa et al., 2019).

### Neural Stem Cell Culture

E14.5 embryonic cortices were dissociated, fragmented and digested with papain (10 U/ml, Worthington Biochemical) and cysteine (1 mM, Sigma-Aldrich) in HBSS with 0.5 mM EDTA at 37°C. The obtained NSCs were routinely cultured in suspension as neurospheres. Cells were normally cultured in neural-inducing medium (NIM) containing: DMEM/F12 (Sigma-Aldrich) supplemented with Hormon Mix (DMEM/F12, 0.6% Glucose (Sigma-Aldrich) [30% in phosphate buffer (PBS) (Euroclone)], Insulin (Sigma-Aldrich) 250 μg/ml, putrescine powder (Sigma-Aldrich) 97 μg/ml, apotransferrin powder (Sigma Aldrich), sodium selenite 0.3 μM, progesterone 0.2 μM), 1 mg/ml penicillin/streptomycin (Sigma-Aldrich), 2 mM glutamine (Sigma-Aldrich), 0.66% Glucose [30% in phosphate buffer salt (PBS) (Euroclone)], Heparin 4 μg/ml, 10 ng/ml basic fibroblast growth factor (bFGF) (Thermo Fisher Scientific) and 10 ng/ml epithelial growth factor (EGF) (Thermo Fisher Scientific). To assess multipotent differentiation capacity of NSCs, cells were seeded on matrigel (Corning) coated glass coverslips at a density of 3–4 × 10^5^ cells per well in a 24 multi-well plate. The first day after plating, the medium was changed adding normal NIM without EGF for 2 days. The medium was then changed again adding NIM without both EGF and FGF, supplemented with 2% fetal bovine serum (FBS) (Sigma-Aldrich) for 6 days.

NSCs proliferation rate was calculated by seeding 6 × 10^5^ cells in adherent conditions in a 6 multi-wells plate and counting (after detaching) the cells every 2–3 days. After the count, 6 × 10^5^ cells were seeded again for 6 time points (3 replicates for each time point).

### Quantitative RT-PCR

RNA was extracted using TRI Reagent (Merck) according to the manufacturer’s instructions. Quantitative RT-PCR (qRT-PCR) was performed as previously described (Sessa et al., 2019) with custom designed oligos (Supplementary Table S1).

### Immunocytochemistry

Immunocytochemicalstaining was performed as previously described (Sessa et al., 2019).

The primary antibodies utilized were the following: anti-KI67 (Immunological Science, MAB90948), anti-GFAP (Merck, AB5804), anti-TUJ1 (Covance, MRB-435P), anti-phospho histone 3 (PH3) (rabbit, 1:400, Sigma-Aldrich, H0412), anti-S100b (Dako, GA504). Anti-O4 primary antibody was produced from a hybridoma clone, using the culture media of hybridoma cells directly on living cells to perform the staining for O4 epitope. Secondary antibody used: 488-mouse (donkey, 1:2,000, Molecular Probes, A21202), 488-rabbit (donkey, 1:2,000, Molecular Probes, A21026), 594-mouse (donkey, 1:2,000, Molecular Probes, A10036), 594-rabbit (donkey, 1:2,000, Molecular Probes, A21207). DAPI (4′,6′-diamidino-2-phenylindole) was used as nuclear counterstaining.

The quantification of Radial Glia cells in the hippocampus has been performed using GFAP antibody, counting only the GFAP^+^ cells present in the subgranular zone of the dentate gyrus.

### RNA-Sequencing

RNA libraries were generated, quality-checked and sequenced as previously described (Sessa et al., 2019). Sequences (Fastq files) were aligned to the mm9 and mm10 mouse reference genomes by using the splice junction mapper TopHat (Kim et al., 2013). Differential gene expression and Functional enrichment analyses were performed with DESeq2 (Love et al., 2014) and GSEA (Huang et al., 2009), respectively. Statistical and downstream bioinformatics analyses were performed within the R environment. The software Homer was used to find *de novo*-enriched motifs in the promoters of downregulated genes with the following setting: +1,000–100 from TSS. The data were deposited in the NCBI Gene Expression Omnibus and are accessible through GSE162750. RNA-seq and ChIP-seq data from the literature were downloaded from the NCBI GEO repository with the accession codes GSM935653, GSM1817009, and GSM1817010.

### Constructs and NSC Complementation

The coding sequence of *Tbl1xr1* was kindly provided by Dr. V. Perissi and cloned into pCAG-V5 vector after digestion with *Xho*I/*Not*I enzymes. Then, Tbl1xr1-V5 coding sequence was amplified using specific primers (5′-TCCCCCCGGGATGAGTATAAGCAGTGATGAGGTCAACT TCTTGG-3′; 5′-ACGCGTCGACTCACGTAGAATCGAGACC GAGGAGAGGG-3′), and inserted in the Ef1a-Setd5-V5 lentiviral construct digested with Xma1 and Sal1 to remove the Setd5 coding sequence and obtain the Ef1a- Tbl1xr1-V5 lentiviral construct. Ef1a-Tbl1xr1-V5 was subjected to PCR site-specific mutagenesis to obtain the following mutated form: (i) Ef1a- Tbl1xr1F10L-V5 (5′-CTCGAGGGATCCACCATGAGTATAAGCAGTGATGAGGT CAACTTgTTGGTATATAGGTACTTGCAAG-3′; 5′-CGGCTG CATGCTGCTGTGCAAGCTTGTCTCTGTAGGCTTGTTGTC TTGTTTGGACTACATCGGGCATAACAGC-3′): (ii) Ef1a- Tbl 1xr1G70N-V5 (5′-CTCGAGGGATCCACCATGAGTATAAG CAGTGATGAGGTCAACTTCTTGGTATATAGGTACTTGCA AG-3′; 5′-GTTGTCTTGTTTGGACTACATCGGGCATAACA GCATCTATCAGGGACAGAGACTCGATGGGTCGACCATCA AATAAGGTGtCATCCTCATTTATGCTAAC-3′); (iii) Ef1a- Tbl 1xr1L282P-V5 (5′-CTTGCCAGCACCTTGGGGCAGCATAAA GGTCCTATATTTGCATTAAAATGGAATAAGAAAGGAAAT TTCATCCCAAGTGCTGGCGTAGATAAG-3′; 5′-GCGGC CGCGGATCCTTTCCGAAGGTCTAAGACACAAACTGAACC GTCCGAAGCAC-3′); (iiii) Ef1a- Tbl1xr1Y446C-V5 (5′-CTTTGACAAAACATCAAGAGCCCGTGTGCAGTGTGGCT TTTAGTCCTGATGGC-3′; 5′-GCGGCCGCGGATCCTTTCCG AAGGTCTAAGACACAAACTGAACCGTCCGAAGCAC).

The lentiviral construct of both wildtype and mutated forms of Tbl1xr1 were used to infect NSC derived from telencephalic cortex of Tbl1xr1 KO embryos at E14.5.

### Immuno-Precipitation

HEK293 cells were seeded on six 150 mm dishes and transfected with Ef1a-GFP (mock), Ef1a-Tbl1xr1-V5, Ef1a-Tbl1xr1F10L-V5, Ef1a-Tbl1xr1 G70N-V5, Ef1a-Tbl1xr1L282P-V5 and Ef1a-Tbl1xr Y446C-V5 using Ca-phosphate. 48 h after transfection, cells were harvested and suspended in 500 μl of IP buffer (20 mM Tri-HCl pH 7.5, 150 mM NaCl, 1 mM EDTA, 1 mM EGTA, 1% Triton X-100) supplemented with complete protease inhibitors (Roche). 30′ after the incubation on ice, the lysate was centrifuged at 14,000 × *g* for 10 min at 4°C and 15% of the supernatant was added with 4X SDS protein sample buffer [100 mM Tris-HCl pH 6.8, 40% (v/v) glycerol, 312 mM SDS, 174 mM dithiothreitol (DTT), 0.04% (w/v) bromophenol blue] (input). The remaining supernatant was immuno-precipitated with V5-antibody overnight at 4°C with agitation. 100 μl of Protein G Dynabeads (Novex, Thermo Fisher Scientific) were used per IP. After washing in IP buffer supplemented with protease inhibitors, the beads were mixed with lysate and incubated for 2 h at 4°C with agitation. At the end of the incubation, the mixed beads-lysate was washed 3 times with IP buffer and added with 2X SDS protein sample buffer for Western blot analysis.

### Quantification and Statistical Analysis

Data are expressed as mean ± standard error (SEM) and significance was set at *p* < 0.05. Statistical tests are provided in the figure legends.

## Results

### *Tbl1xr1* Knock-Out Mice Display Behavioral Impairments

To analyze the importance of TBL1XR1 for neurological capabilities in living animals, we generated *Tbl1xr1* knock-out (KO) mice taking advantage of a line carrying the *Tbl1xr1* floxed allele (LoxP sequences surrounding the exon V) (Rohm et al., 2013) crossed with the CMV:Cre driver line (Figure 1A). Despite the full KO mouse died at an early embryonic phase (Perissi et al., 2004), CMV:Cre mediated KO animals were born nearly at mendelian ratio, successfully completed pre-natal development and reached the adulthood (Figure 1A), perhaps thanks to incomplete DNA recombination at some stage/cell population. However, the TBL1XR1 protein was completely absent in brain tissue (Figure 1A). Since mutations in the *TBL1XR1* are linked with a variety of neuro-psycho-motor disabilities, we decided to test motor, social, and cognitive performance of KO vs. control littermate mice through behavioral assessment (Figure 1 and Supplementary Figure S1). Both genotypes showed comparable spontaneous activity in the open field (Supplementary Figure S1A), while motor coordination was impaired in KO mice as revealed by poor performance in rotarod exercise (Figure 1B), abnormal paw-slipping in beam walking (Figure 1C), and mild but appreciable differences in some catwalk parameters (Figure 1D and Supplementary Figure S1B). Interestingly, a large fraction of human *TBL1XR1* mutated patients display compromised or delayed motor skills (Vaqueiro et al., 2018).

Mutant animals tended to hide more objects compared to the WT in the marble-burying test, possibly reflecting obsessive behavior, and/or anxiety (Figure 1E). However, the time spent at the periphery vs. the center of an empty arena was comparable, indicating that *Tbl1xr1* KO did not show high anxiety levels (Supplementary Figure S1C). We also performed an elevated plus maze test to further investigate anxiety. Notably, mutant mice did not display significant differences in the time spent in the open arms, confirming that the loss of *Tbl1xr1* is not impacting on anxiety, at least in this model (Supplementary Figure S1D).

Next, we assessed behavior that is relevant for intellectual disability and autism spectrum disorders. To test spatial memory, we used an 8-arm radial maze test (Figure 1F), in which *Tbl1xr1* mutants displayed significant differences in memory performances, including working memory (Figure 1F and Supplementary Figure S1E). Sociability was assayed by evaluating the time that a test subject (either a WT or an KO animal) spent together with a host WT mouse (Silverman et al., 2010). KO animals interacted significantly less with the host, compared to the controls (Figure 1G). However, the two genotypes spent equal amount of time in aggressive behavior (Supplementary Figure S1F). The animals did not present any olfactory impairment, since both groups performed food tests (either hidden or presented) equally well (data not shown). Furthermore, since external factors may confound the assessment of sociability in freely moving subjects, we performed a three-chamber test (Papale et al., 2017) where the stranger mouse is confined in a cage (thus eliminating any possible effect of size or dominance) while the subject *Tbl1xr1* KO or WT animals was free to explore. Despite the two genotypes equally examined the arena (Supplementary Figure S1G), mutant animals did not discriminate between the stranger-occupied cage and the empty one (Figure 1H). Notably, cognitive disability is a penetrant trait in TBL1XR1 patients, while low sociality is present in TBL1XR1 patients with ASD (Laskowski et al., 2016; Vaqueiro et al., 2018).

These results indicate that our mouse model, in which TBL1XR1 is completely missing in brain tissue, is viable and presents behavioral deficits that, at least in part, resemble the impairments described in patients carrying genomic insults in the *TBL1XR1* gene, thus representing an interesting model for the related diseases.

### Loss of *Tbl1xr1* Alters Morphology and Function of Neuronal Cells

**FIGURE 2:**
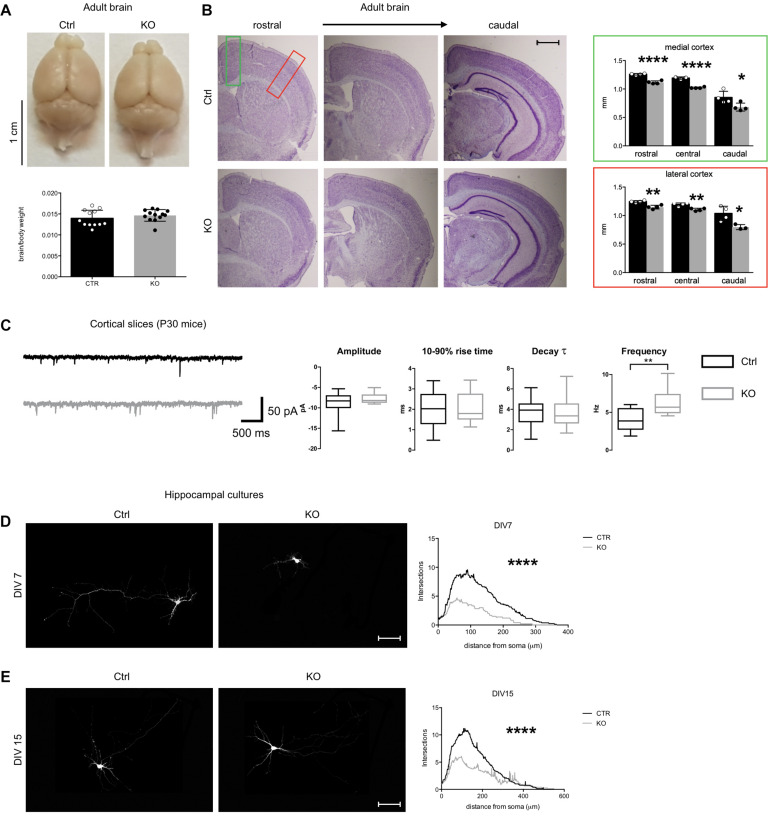
Defects of *Tbl1xr1* mutant neurons. **(A)** Adult brains from both control and Tbl1xr1 mutant mice showed similar brain to body ratio (shown as means ± SEMs, with dots representing individual samples), *n*: Ctrl = 12, KO = 13: *p* > 0.05, unpaired *t*-test. **(B)** Left, cresyl violet staining of coronal section of both control and mutant forebrains (cerebral corte, striatum, and hippocampus from adult mice of >than 2 months of age). right quantification of the cortical thickness in medial (green square) and lateral (red) position (shown as means ± SEMs, with dots representing individual samples), *n*: Ctrl = 4, KO = 4: rostral: ****p < 0.0001; central: ****p < 0.0001; rostral: *p < 0.0415. **(C)** Left, representative traces showing sEPSCs recorded in cortical pyramidal neurons in slices from control (black) and *Tbl1xr1* mutant (gray) mice (animals of both sexes, 30 days of age) (bars: 50 pA, 500 ms). Right, summary boxplots for EPSC parameters (shown as box for interquartile range, median line and whiskers for highest and lowest values): mean amplitude, wt -8.6 ± 0.6 pA, KO -7.8 ± 0.3 pA, *n* = 19, *p* = 0.27, unpaired *t*-test; mean 10–90% rise time, wt 1.9 ± 0.2 ms, KO 2.0 ± 0.2 ms, *n* = 19, *p* = 0.78, unpaired *t*-test; mean decay time constant, wt 3.7 ± 0.3 ms, KO 3.6 ± 0.4 pA, *n* = 19, *p* = 0.87, unpaired *t*-test; mean frequency, wt 4.0 ± 0.3 Hz, KO 6.3 ± 0.4 Hz, *n* = 19, *p* < 0.001, unpaired *t*-test. **(D)** Hippocampal cultures from control and mutant E17.5 embryos at 7 days *in vitro* (DIV) infected with low titer GFP lentivirus (at DIV0) and Sholl analysis for the quantification of the intersection of neurites accordingly with the distance to the soma, ****p < 0.0001, 2-way ANOVA. **(E)** Hippocampal cultures from control and mutant E17.5 embryos at 14 days *in vitro* (DIV) infected with low titer GFP lentivirus (at DIV0) and Sholl analysis for the quantification of the intersection of neurites accordingly with the distance to the soma, ****p < 0.0001, 2-way ANOVA. Scale bars: **(B)** 400 μm; **(D,E)** 50 μm. See also Supplementary Figure S2.

Mutant brains appeared grossly normal in the adulthood, and the brain weight normalized to total body mass was conserved (Figure 2A). However, cortical wall thickness was reduced, particularly at medial-caudal level (Figure 2B). Although neuronal types were conserved across the cortical layers, we found that the number of CUX1^+^ late-born neurons was significantly higher and SATB2^+^ neurons were decreased in mutants, while the number of the early born CTIP2^+^ neurons was unaltered (Supplementary Figure S2A). Thus, the molecular identity of upper layer neurons (where both CUX1^+^ and SATB2^+^ neurons are particularly abundant) is altered in these mutants.

To determine if synaptic transmission was impaired upon loss of *Tbl1xr1*, we analyzed both spontaneous glutamatergic excitatory postsynaptic currents (sEPSCs) and GABAergic inhibitory postsynaptic currents (sIPSCs) in cortical slices from postnatal day 30 (P30) KO and littermate WT mice using whole-cell voltage-clamp recordings (Figure 2C). While amplitude, 10–90% rise time, and decay time constant were unaltered, the sEPSC frequency was significantly increased in mutant neurons (Figure 2C). Conversely, we found no differences in sIPSC parameters between genotypes (data not shown). Then, we sought to further investigate neuronal cells using primary neuronal cultures from hippocampi of E17.5 mutant and WT embryos. Mutant neurons exhibited a simpler morphology, with less neurites and arborization compared to control cells both at early and late stages of *in vitro* development (Figures 2D,E). In developing mutant neurons (DIV 15), dendritic spines were significantly sparser than controls (Supplementary Figure S2B), though their number was comparable in more mature cells *in vitro* (Supplementary Figure S2C). Golgi-Cox staining confirmed the normal dendritic spine number in adult KO cerebral cortex compared to controls, without evident changes in their shape (Supplementary Figure S2D).

Altogether, these results suggest that *Tbl1xr1* mutant mice are mildly microcephalic, as seen in particular in Pierpont syndrome patients (Heinen et al., 2016), and that their excitatory synapses are hyperactive.

### Loss of *Tbl1xr1* Leads to Defective Proliferation and Differentiation of Neural Progenitors

**FIGURE 3:**
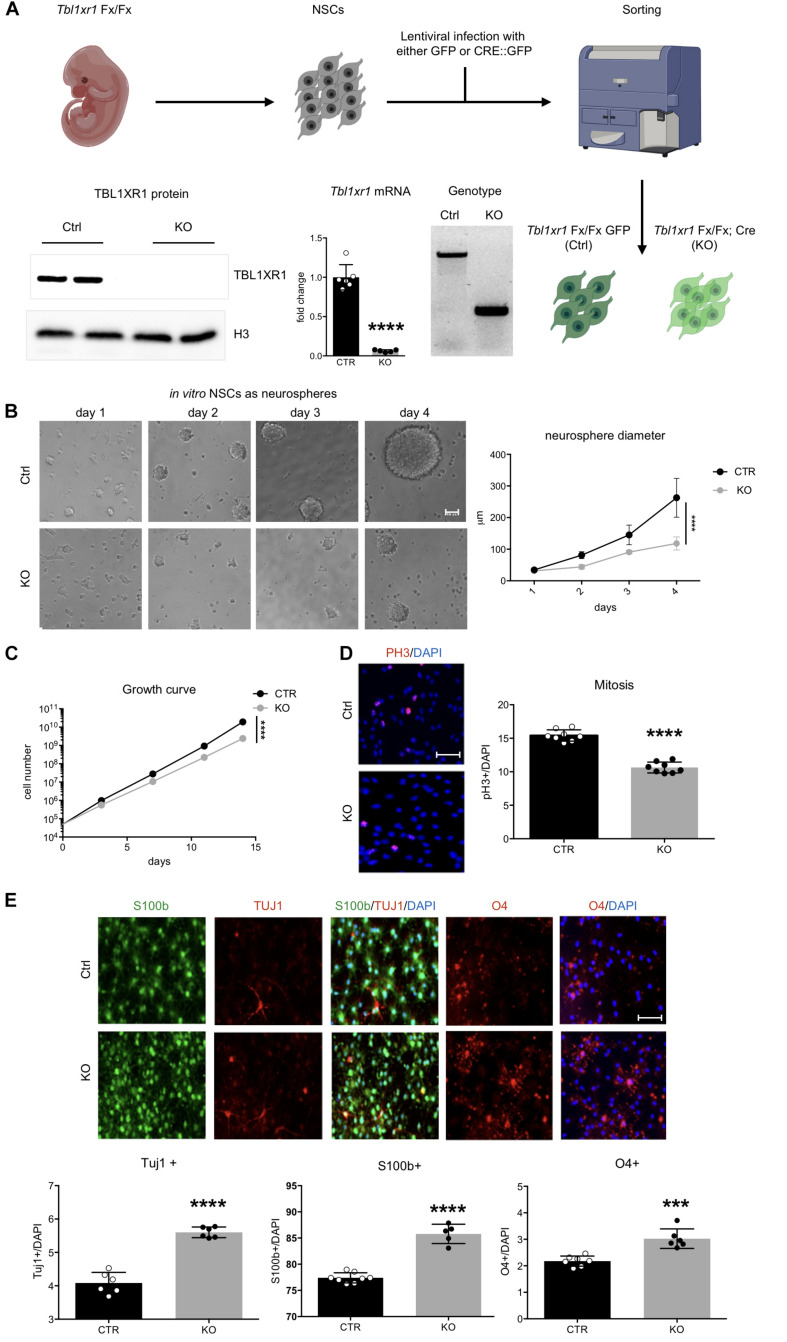
Loss of *Tbl1xr1* affects neural stem cells dynamics. **(A)** Scheme of NSC derivation with examples of: genotyping (by PCR), expression of *Tb1xr1* (mRNA abundance by RT-qPCR, ****p < 0.0001, unpaired *t*-test), and TBL1XR1 protein level (by Western blot). **(B)** NSCs both Ctrl and KO cultured as neurospheres, on the right quantification of spheres’ diameter: difference due to genotype, *F*(1,40) = 63.19, ****p < 0.0001, 2-way ANOVA. **(C)** Growth curve of adherent *in vitro* NSCs: difference due to genotype, *F*(1,4) = 535.6, ****p < 0.0001, 2-way ANOVA. **(D)** Immunocytochemistry of both Ctrl and *Tbl1xr1* KO proliferating NSCs for phosphor histone 3 (PH3) counterstained with DAPI. On the right, quantification of PH3^+^ cells on total DAPI nuclei, ****p < 0.0001, unpaired *t*-test. **(E)** Up, immunocytochemistry of both Ctrl and *Tbl1xr1* KO differentiating NSCs for S100b (astrocytes), TUJ1 (neurons) and O4 (oligodendrocytes) counterstained with DAPI. Bottom, quantification: TUJ1: ****p < 0.0001, unpaired *t*-test; S100b: ****p < 0.0001, unpaired *t*-test; O4: ***p = 0.0002, unpaired *t*-test. Scale bars: **(B)** 40 μm; **(D,E)** 50 μm.

We next sought to investigate whether the observed changes reflected any impairment in brain development during embryonic life. The embryonic cortex at E14.5 was slightly smaller in mutants, particularly in its medial region (Supplementary Figure S3A). Interestingly, the ventricular zone of mutant mice contained less proliferative progenitors (PAX6^+^ cells), probably because differentiation occurred at earlier stages (SATB2^+^ used as marker of young neuronal cells) (Supplementary Figure S3B). To better analyze neural progenitor proliferation/differentiation dynamics, we used an *in vitro* system constituted by NSCs (Azari et al., 2010). To avoid possible residual early stage impairments, we derived NSCs from *Tbl1xr1* floxed E14.5 embryos. After infecting them with lentivirus carrying either Cre-GFP (to generate KO’s) or GFP only (as control), we sorted the GFP^+^ cells and verified for correct genotypes and for Tbl1xr1 mRNA and protein absence (Figure 3A). When grown as neurospheres, mutant NSCs showed a smaller sphere volume compared to controls (Figure 3B), possibly due to a proliferative defect. When we alternatively used adherent cultures to obtain proper growth curves and immuno-labeling (using PH3 and KI67 proliferative markers), a decrease in cell cycle progression was evident (Figures 3C,D and Supplementary Figure S3C). Conversely, the differentiation capability of mutant NSCs were increased since KO cells generated more post-mitotic TUJ1^+^ neurons, S100b^+^ astrocytes, and O4^+^ oligodendrocytes after applying a differentiation protocol *in vitro* (Figure 3E).

Finally, to investigate whether these defects are present in *Tbl1xr1* mutant postnatal progenitors, we analyzed the dentate gyrus (DG) of the hippocampus where adult neurogenesis normally occur in rodents. First, we noted that the gross morphology of the hippocampus and the DG in particular is maintained (Figure 2B). Immunochemistry for markers of radial glia cells (GFAP), neuroblasts/immature neurons (DCX), neurons (NEUN), and proliferative cells (KI67), indicated that the dynamics of proliferation/differentiation in both mutant and control DG is similar (Supplementary Figure S3D).

These data indicate that the loss of TBL1XR1 during the neural progenitor stage leads to proliferative defects and a tendency to anticipate differentiation that may eventually induce a microcephalic brain. However, adult neurogenesis in DG is preserved in *Tbl1xr1* mutants.

### TBL1XR1 Ensures Correct MAPK Signaling Transduction

**FIGURE 4:**
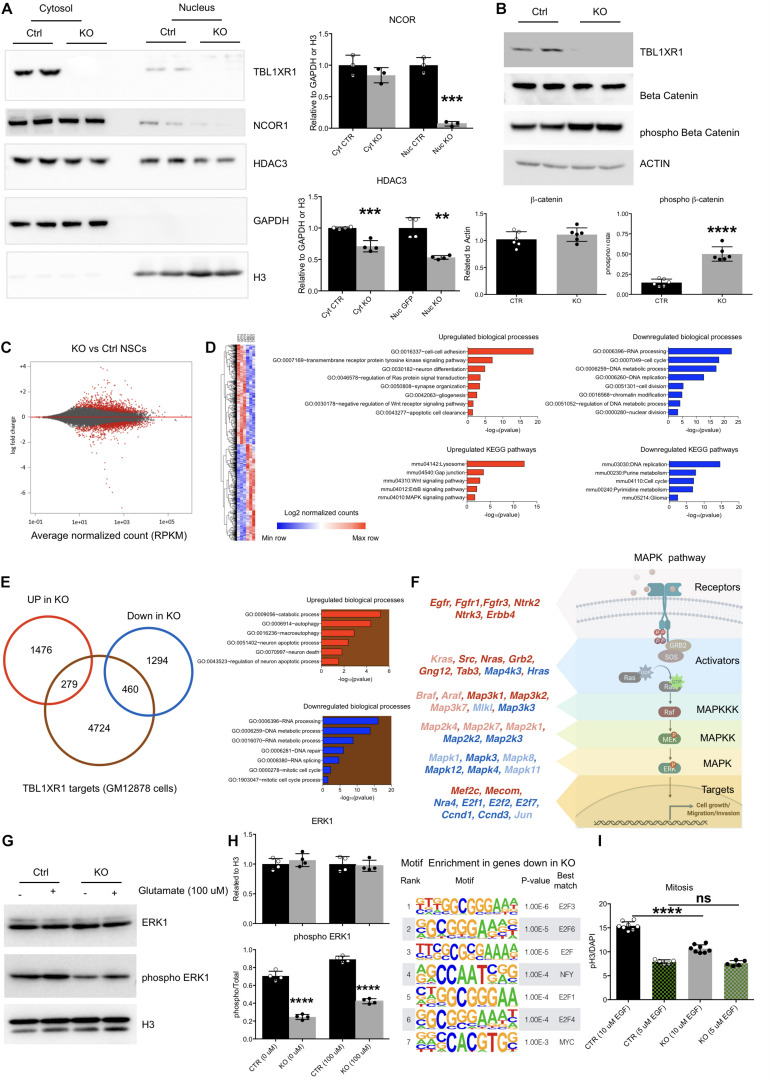
Molecular defects of *Tbl1xr1* KO neural stem cells. **(A)** Western blot analysis of both cytosolic (enriched in GAPDH, used as normalizer) and nuclear (enriched in H3, used as normalizer) protein fractions from control and *Tbl1xr1* KO *in vitro* NSCs for the following proteins: TBL1XR1, NCOR1, and HDAC3. On the right, the quantification of the blots for NCOR1 and HDAC3 (shown as mean + s.e.m. with dots representing individual samples): cytoplasmic NCOR1: n (biological replicates): Ctrl = 3, KO = 3: *p* = 0.2407; nuclear NCOR1: *n* (biological replicates): Ctrl = 3, KO = 3: ***p = 0.0002; cytoplasmic HDAC3: *n* (biological replicates): Ctrl = 4, KO = 4: ***p = 0.0007; nuclear HDAC3: *n* (biological replicates): Ctrl = 4, KO = 4: **p = 0.0013. Statistically compared using *t*-test. **(B)** Western blot analysis of whole protein lysates from control and *Tbl1xr1* KO *in vitro* NSCs for the following proteins: TBL1XR1, βCATENIN and its phosphorylated form (Ser33/37/Thr41). On the right, the quantification of the blots for βCATENIN and pβCATENIN: *n* (biological replicates): Ctrl = 6, KO = 6: βCATENIN *p* = 0.2878; pβCATENIN ****p < 0.0001. Statistically compared using unpaired *t*-test. **(C)** MA plot showing log2 fold changes as function of average normalized gene expression (RPKM) for control against KO *in vitro* NSCs (RNAseq data). Differentially expressed genes are highlighted in red. **(D)** Left, heat-map showing the differentially expressed genes between control and KO NSCs. Right, Gene Ontology analysis for terms indicating both the biological processes (up) and KEGG pathways (bottom), for upregulated (red, left) and downregulated (blue, right) genes in *Tbl1xr1* KO NSCs compared to control. **(E)** Left, Venn diagram showing the overlap between TBL1XR1 gene targets (brown, publicly available ChIP-seq data obtained from GM12878 cells) and genes up (red) and down-regulated (blue) in *Tbl1xr1* KO NSCs. Right, Gene Ontology analysis for terms indicating the biological processes for TBL1XR1 targets that are also upregulated (the red/brown intersection) and downregulated (the blue/brown intersection) genes in *Tbl1xr1* KO NSCs compared to control. **(F)** MAPK pathways found altered between control and *Tbl1xr1* KO NSCs ordinated accordingly their role within the pathway (scheme on the right). Red = upregulated in mutant; blue = downregulated in mutant; dark color = statistically significant; light color = trend. **(G)** Western blot analysis of whole protein lysates from control and *Tbl1xr1* KO NSCs treated or not with 100 μM of glutamate for the following proteins: ERK1 and its phosphorylated form (Thr202/Tyr204). On the right, the quantification: *n*: Ctrl = 4, KO = 4, 0 mM of glutamate: ERK1 *p* = 0.3898; pERK1 ****p < 0.0001; 100 μM of glutamate: ERK1 *p* = 0.7170; pERK1 ****p < 0.0001; Statistically compared using unpaired *t*-test. **(H)** Motif enrichment analysis using Homer on promoters (–1,000 + 100 from TSS) og genese downregulated in Tbl1xr1 KO NSCs (*n* = 1,754). **(I)** Quantification of the immunocytochemistry for PH3 in both Ctrl and *Tbl1xr1* KO proliferating NSCs in a medium with either normal (10 μM) or low levels (5 μM) of EGF, counterstained with DAPI. 10 μM: n: Ctrl = 8, KO = 8, ****p < 0.0001; *n*: Ctrl = 5, KO = 5, *p* = 0.9055; one-way ANOVA. See also Supplementary Tables S2, S5.

Together with its related protein TBL1X, TBL1XR1 is important for both gene repression mediated by NCOR/SMRT complexes, and for de-repression promoting the removal of NCOR/SMRT (Perissi et al., 2010). Thus, we investigated the level of NCOR1 using Western blot analyses on both NSCs and adult brains. The level of NCOR1 was decreased in mutants, indicating that TBL1XR1 is critical for the assembly of the co-repressor complex rather than serving as an exchanging factor (Supplementary Figures S4A,B). The entire complex seemed to be affected since also HDAC3, the typical histone deacetylase present in the complex, was significantly downregulated both *in vitro* and *in vivo* (Supplementary Figures S4A,B). The effect on both NCOR1 and HDAC3 was particularly evident in the nuclear fraction, where the epigenetic complex is normally operating (Figure 4A). Both TBL1R and TBL1XR1 are also required for transcriptional activity of βCATENIN on Wnt target genes (Li and Wang, 2008). Interestingly, despite the level of βCATENIN was unaffected, we detected a high level of its phosphorylated form in the mutant cells (Figure 4B) suggesting that, in the absence of TBL1XR1, the AXIN2/GSK3β complex is active to remove the protein and mitigate the canonical Wnt signaling (MacDonald et al., 2009).

To gain insights on the molecular aspects of the phenotype, we performed RNA-seq to analyze the global transcriptome upon *Tbl1xr1* loss. Approximately 3,500 transcripts were de-regulated (1,755 upregulated and 1,754 downregulated) (Figure 4C and Supplementary Table S2). Gene Ontology (GO) analysis showed that several biological processes were significantly altered in *Tbl1xr1* KO cells, including DNA replication and cell cycle (downregulation), as well as gliogenesis and neuron differentiation (upregulation) (Figure 4D and Supplementary Table S2), confirming our previous observations (Figure 3). Moreover, we found upregulated gene categories associated with important molecular pathways as the Wnt and MAPK pathways, while several categories associated with nucleic acid metabolism were downregulated (Figure 4D and Supplementary Table S2). To discriminate between possible direct and indirect effects of TBL1XR1 on gene transcription, we examined public available datasets of DNA regions bound by TBL1XR1 obtained in a lymphoblastoid cell line (GM12878 cells). Despite the cell origin was very far from NSCs, we observed that a significant percentage of both upregulated (16%) and downregulated genes (26%) are putative TBL1XR1 direct targets (Figure 4E). These target genes account for GO categories important for DNA/RNA catabolism, RNA processing and splicing, DNA repair, and cell cycle (putative direct targets downregulated) as well as catabolism, autophagy and cell death (putative direct targets upregulated) (Figure 4E and Supplementary Table S2). These observations support the double nature of the TBL1XR1 protein to serve both as a core member and an exchanging factor of NCOR complexes, explaining the missing repressive activity on certain loci and the transcriptional repression on others, respectively, in *Tbl1xr1* KO’s. However, it has to be noted that the paralog gene *Tbl1x* was upregulated in *Tbl1xr1* NSCs (Supplementary Table S2), possibly vicariating the function of TBL1XR1 in certain contexts.

To understand the impact of the lack of TBL1XR1 on Wnt signaling, we analyzed direct target genes of this pathway that resulted partially deregulated. We found that transcripts changed in both directions in our dataset, that did not agree with a loss of βCATENIN activity (Supplementary Figure S4C and Supplementary Table S3). Next, we treated both control and KO NSCs with either an activator (LiCl) (Clément-Lacroix et al., 2005) or an inhibitor (IWR-1) (Martins-Neves et al., 2018) of the Wnt pathway. Interestingly, both genotypes were competent to increase cell proliferation, reaching the same level upon the activation of the pathway (Supplementary Figure S4D). However, the inhibition of βCATENIN, although affecting the proliferation of both lines, did not blunt the difference between control and KO NSCs (Supplementary Figure S4E). These data suggest that other pathways beyond Wnt contribute to the proliferative defects of *Tbl1xr1* KO NSCs.

The NCOR complex intersects multiple signaling cascades (Perissi et al., 2010). Our transcriptomic data showed a heavy de-regulation of the mitogen-activated protein kinase pathway. GO analysis indicated an upregulation of the MAPK pathway (Figure 4D), which are particularly relevant for the upper part of the cascade (e.g., receptors and MAP3K1 and 2), while other genes belonging to downstream nodes (e.g., transcription factors) were mostly downregulated (Figure 4F, Supplementary Figures S4F,G, and Supplementary Table S4). Of note, both NCOR1 and NCOR2 appear to directly target several MAPK-related genes (Supplementary Figures S4H,I and Supplementary Table S4). Thus, we decided to investigate whether this pathway is affected in our system. First, we biochemically evaluated the phosphorylated form of the extracellular signal-regulated kinase 1 (ERK1) as a proxy for its general activation, showing a strong decrease in *Tbl1xr1* KO NSCs both in basal condition and upon exogenous stimulation (100 μM glutamate) (Figure 4G). Notably, the general level of the protein was maintained (while the mRNA level was downregulated, Figure 4F), indicating that the alteration can be either subtle (and thus not detected in whole protein lysates) or recovered at post-transcriptional levels.

Then, we found that the genes downregulated in *Tbl1xr1* KO NSCs were enriched in E2F binding motifs near their promoters (−1,000 + 100 bp from the TSS) (Figure 4H, Supplementary Figure S4J, and Supplementary Table S5). Importantly, genes encoding for these factors such as *E2f1* and *E2f2*, known as MAPK downstream effectors (Nikolai et al., 2016), were downregulated upon *Tbl1xr1* loss (Supplementary Figure S4K).

NSCs *in vitro* proliferation relies on the external supply of mitogens, which eventually converge on MAPK cascade (Galli et al., 2003). Thus, we decreased the concentration of one of these factors (EGF) by 50% in the culture medium of both WT and KO cells. The reduced availability of EGF blunted the proliferative difference between the two genotypes, suggesting that the transduction of mitogen-activated cascade is affected by the loss of TBL1XR1 in NSCs (Figure 4I).

Altogether, these results suggest that depletion of TBL1XR1 affects directly and indirectly a number of important intracellular pathways. Among these, a prominent role is played by the MAPK pathway branch that is activated by external stimuli like mitogens. In fact, despite a de-repression of the upstream portion, the MAPK cascade results partially blocked leading to proliferation defects through the impairment of E2F transcription factors target network.

### MAPK Signaling Alteration Is a Differential Feature Between *TBL1XR1*-Associated Disorders

**FIGURE 5:**
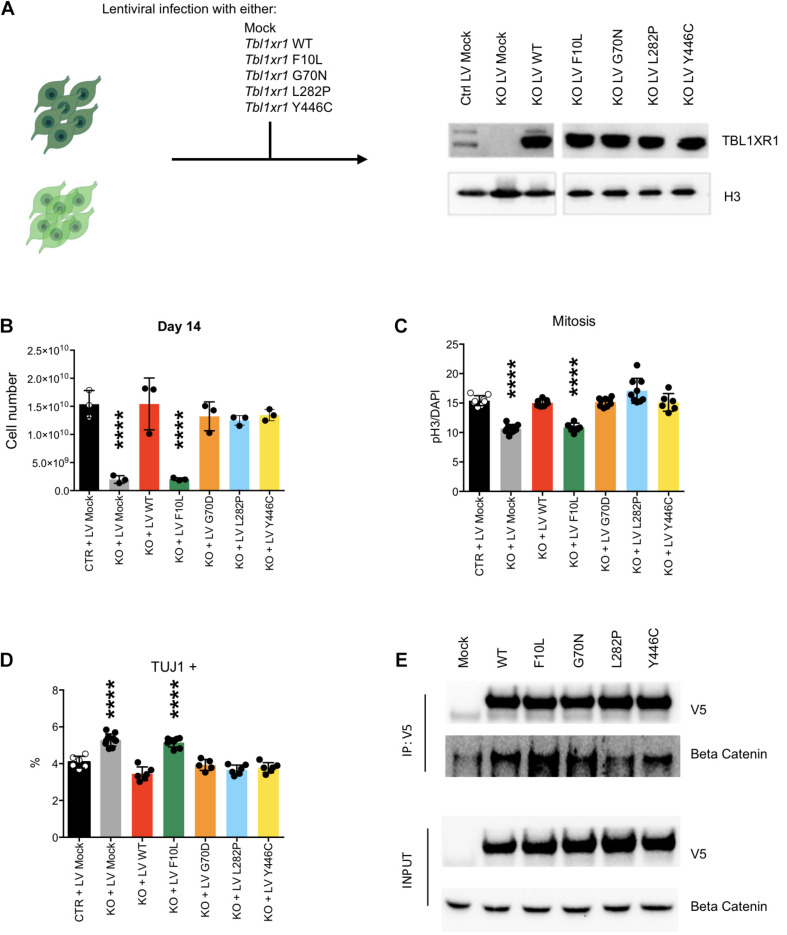
Complementation of *Tbl1xr1* KO neural stem cells. **(A)** Experimental design and Western blot analysis of whole protein lysates from the indicated NSCs for TBL1XR1 (H3 was used for normalization). **(B)** Final point (day 14) of growth curves of the indicated NSCs (see also Supplementary Figure S5A) (shown as mean + s.e.m. with dots representing individual samples): Ctrl + LV Mock vs.: KO + LV Mock ****p < 0.0001; KO + LV WT *p* > 0.9999; KO + LV F10L ****p < 0.0001; KO + LV G70D *p* = 0.7111; KO + LV L282P *p* = 0.4644; KO + LV Y446C *p* = 0.7894; one-way ANOVA with Dunnett’s multiple comparisons test. **(C)** Quantification of the immunocytochemistry for PH3 in the indicated NSCs (see also Supplementary Figure S5C) (shown as mean + s.e.m. with dots representing individual samples): Ctrl + LV Mock vs.: KO + LV Mock ****p < 0.0001; KO + LV WT *p* = 0.9710; KO + LV F10L ****p < 0.0001; KO + LV G70D *p* = 0.9960; KO + LV L282P *p* = 0.0556; KO + LV Y446C *p* = 0.9924; one-way ANOVA with Dunnett’s multiple comparisons test. **(D)** Quantification of the immunocytochemistry for TUJ1 in the indicated NSCs (shown as mean + s.e.m. with dots representing individual samples): Ctrl + LV Mock vs.: KO + LV Mock ****p < 0.0001; KO + LV WT *p* = 0.1075; KO + LV F10L ****p < 0.0001; KO + LV G70D *p* = 0.8997; KO + LV L282P *p* = 0.0789; KO + LV Y446C *p* = 0.3241; one-way ANOVA with Dunnett’s multiple comparisons test. **(E)** Western blot analysis of precipitated fraction and input of immune-precipitation experiment using the antibody against V5 tag that is fused to each Tbl1xr1 isoforms, for V5 and βCATENIN.

To investigate the effects of different disease-inducing *TBL1XR1* mutations, we designed a series of complementation experiments using *Tbl1xr1* KO NSCs and lentiviral constructs carrying either *Tbl1xr1* WT coding sequence or its mutated versions corresponding to the following human mutations: F10L (case of schizophrenia) (Nishi et al., 2017), G70N (West syndrome-like) (Saitsu et al., 2014), L282P (ASD) (O’Roak et al., 2012) and Y446C (Pierpont syndrome) (Pons et al., 2015; Figure 5A). All versions were expressed at supra-physiological levels in NSCs (Figure 5A). The *Tbl1xr1* WT form was able to restore the proliferation defects of KO NSCs as shown by (i) the growth curve (Supplementary Figures S5A,B), (ii) the dimension of the neurospheres, when the cells were cultures as free-floating in the media (Supplementary Figure S5B) and (iii) the quantification of the immunostaining for M-phase marker (Figure 5C and Supplementary Figure S5C). In addition, the tendency to differentiate faster in post-mitotic derivatives was counteracted by lentiviral mediated *Tbl1xr1* expression (Figure 5D and Supplementary Figure S5D). This suggests that the defects we reported are specifically due to TBL1XR1 absence and that a fast re-introduction of the factor completely reverts the phenotype. The different mutations we tested were all equally able to rescue both the proliferative capacity and the normal differentiation, except for F10L, which failed any recovery (Figures 5B–D and Supplementary Figure S5). Also, G70N displayed an incomplete reversion of neurosphere diameter (Supplementary Figure S5B) but showed a normal rate of proliferation and differentiation (Figures 5B–D and Supplementary Figure S5). This suggests that all the missense mutations considered, except F10L, do not rely on a loss-of-function mechanism. Conversely, the schizophrenia phenotype due to F10L mutation may be due, at least in part, by endophenotypes similar to those shown by the KO. It has been reported that F10L substitution increased the ability of TBL1XR1 in the transduction of Wnt signaling, while the association with NCOR complex seemed negatively affected (Nishi et al., 2017). We independently confirmed that, among the tested TBL1XR1 mutant forms, the F10L was the one with the greater affinity for βCATENIN (Figure 5E). Thus, our complementation experiment supports the hypothesis that the phenotype we observed in KO cells is due to an impairment of NCOR-related functions, rather than Wnt signaling.

Collectively, these data suggest that within the complex mutational spectrum of the *TBL1XR1* gene, some genetic conditions (deletions and F10L) cause early neural defects due to dysregulation of MAPK cascade through the missing interaction with NCOR complex, while for other mutations (e.g., G70N, L282, and Y446C) other mechanisms must be considered.

## Discussion

In this study, we show that the genetic knock-out of *Tbl1xr1* in mice leads to behavioral impairments that, to a certain extent, are similar to those typical of human patients mutated in the same gene. We demonstrated that *Tbl1xr1* mutant brain exhibits morphological and functional alterations originated by an increased proliferation and delayed differentiation of neural stem cells of the embryonic brain. We found impairments in the NCOR1/HDAC containing complex, transcriptional gene alterations, and a deregulation in the MAPK signaling, which are all plausible mechanisms for the disorders associated with TBL1XR1 loss of function.

*TBL1XR1* mutations have been associated with neurological disorders presenting variegated manifestations that may include brain malformations, social difficulties, intellectual disability, developmental delay, learning disability, hearing loss, schizophrenia, and seizures (Kong et al., 2020). This large spectrum of phenotypes is somehow expected since TBL1XR1 may serve as cofactor for multiple functions, including the regulation of chromatin occupancy by NCOR complexes (Perissi et al., 2010), WNT signaling (Choi et al., 2011) and interaction between NCOR1/2 and MECP2 (Kruusvee et al., 2017). However, the precise function of TBL1XR1 in the processes at the basis of these phenotypes has been neither clarified nor experimentally modeled.

Here we show that animals with *Tbl1xr1* loss-of-function displayed behavioral abnormalities. These include motor coordination, memory skills, and social impairments, which are described at different levels in patients with TBL1XR1 deletions, missense mutation, and Pierpont syndrome (Vaqueiro et al., 2018; Kong et al., 2020). Of note, these phenotypes are also overlapping with some disorders due to either *NCOR1/2* or *HDAC3* mutations (Wang et al., 2016; Sajan et al., 2017; Sakaguchi et al., 2018; Iwama et al., 2019; Zhou et al., 2019). An interesting opportunity of comparison is also given by the existing animal models of TBL1XR1 interactors, namely mutants in *Ncor1/2*, *Hdac3*, and *Mecp2*. It is important to stress how neurological functions, including social and intellectual abilities, resulted indeed altered in these animals (Kong et al., 2020). In particular, our model presents memory and social impairment similar to the knock-in murine model carrying mutations in the deacetylase-activating domain of both NCOR1 and 2, that hinder their binding to HDAC3 (Zhou et al., 2019), supporting similarities between TBL1XR1, NCOR1, and NCOR2 loss-of-function. On the contrary, the two models present opposite results regarding anxiety and locomotor coordination (Zhou et al., 2019). However, our model appears different from *Ncor1/2* and *Hdac3 KO*. In particular, given the multifaceted nature of the TBL1XR1 factor, our model is not reflecting only the consequences of the loss of function of NCOR complexes. Indeed, TBL1XR1 is important for both NCOR1 and NCOR2 activity (leading to epigenetic repression) and for its dismissal (leading to gene activation) and consequently for the fine balance between these two processes. Therefore, the impairments found in our model likely depend from the different contexts of TBL1XR1 action (e.g., either the absence or presence of the ligand for nuclear receptors) in a time- and stage-dependent manner.

Here, we show that *Tbl1xr1* KO mice are microcephalic with hyperactive excitatory synapses. This may be due to different dynamics in neuronal maturation and circuit formation and/or homeostasis, even though a normal number of glutamatergic spines are present in the adult mutant neurons. Since we identified a peculiar pattern of spine development (low number at early stage, physiological number in the adult), it remains possible that the normal wiring processes of the cortical areas are altered during development of *Tbl1xr1* mutants, leading to the hyper-excitability once that the correct number of spines is restored. Further functional investigation will clarify in the future the exact role of TBL1XR1 in this respect.

We demonstrated a reduced proliferation of NSCs upon *Tbl1xr1* KO. The TBL1XR1-mediated regulation of both Wnt signaling and NCOR1 complex may contribute to the observed phenotype. Indeed, βCATENIN, of which TBL1XR1 is an interactor complementing its function as a transcription factor, positively regulates proliferation of neural precursors, particularly in embryonic stages (Oliva et al., 2018). Its upregulation leads to a dramatic tangential enlargement of the neural ectoderm (Chenn and Walsh, 2002; Marinaro et al., 2012). On the other hand, the inhibition of the canonical Wnt signaling leads to a diminished proliferation of early neural progenitors and premature differentiation/apoptosis (Holowacz et al., 2011; Bem et al., 2019), while in both late and adult neural stem cells the effect was the opposite (Hirabayashi et al., 2004; Kuwabara et al., 2009). However, our data obtained with embryonic mid-gestation NSCs do not univocally indicate an upregulation of the Wnt pathway. The NCOR1/2 complexes have been studied in the context of neural progenitor proliferation too (Jepsen et al., 2000, 2007; Hermanson et al., 2002). In *Ncor1* KO mice the cerebral cortex is mildly affected, with an apparent increase in neuronal differentiation at E14.5 (Jepsen et al., 2000). NSCs derived from *Ncor1*^–/–^ embryonic brain failed to form colonies, proliferated less *in vitro*, and differentiated under normal culture conditions (Hermanson et al., 2002). The same study suggested that the nuclear localization (hence the possibility to work as epigenetic modifier) is lost upon differentiation, once again supporting the importance of NCOR1 for the proliferative stage (Hermanson et al., 2002). *Ncor1* knock-down has been also used to decrease the proliferative capacity of GBM cell models, which are relatively similar to NSCs (Heldring et al., 2014). *Ncor2*^–/–^ suggested a role for NCOR2 as a regulator of NSC state counteracting Notch activity and retinoic acid-dependent differentiation (Jepsen et al., 2007). Our data support a role of NCOR1 activity for correct NSC proliferation, implying that *Tbl1xr1* and *Ncor1* loss-of-functions lead to convergent molecular dysfunctions and cellular phenotypes. This is further supported by the fact that F10L is the only *Tbl1xr1* mutation unable to restore the proliferative capability in KO NSCs. TBL1XR1 carrying the F10L substitution is known to have a reduced interaction with the NCOR complex, in favor of an enhanced interaction with βCATENIN (Nishi et al., 2017).

We propose that the TBL1XR1-mediated control of proliferation/differentiation dynamics is through NCOR1-mediated regulation of MAPK signaling. This is supported by the fact that *Ncor1* KO NSCs were irresponsive to FGF2, one of the external mitogens able to stimulate the MAPK cascade (Hermanson et al., 2002; Mossahebi-Mohammadi et al., 2020). It has also been shown that the loss of ERK1/2 activity is detrimental for hippocampal neural progenitors *in vivo* (Vithayathil et al., 2015), as occurs in mutant of FGF receptor mutants (Ohkubo et al., 2004). This mitogen-activated pathway is involved in cancer, including the proliferative capabilities of the most aggressive brain cancers as grade IV glioblastomas (Jimenez-Pascual and Siebzehnrubl, 2019). Gain-of-function mutations in the components of RAS/MAPK pathway have been causally connected with developmental disorders (RASopathies) that include Noonan, Cardio-facio-cutaneous, and Costello syndromes (Kim and Baek, 2019). Several neurological abnormalities including neuro-cognitive impairment, macrocephaly, and seizures characterize these diseases to different levels (Rauen, 2013). At least part of these phenotypes may be due to increased proliferation of mutant neural progenitors (Rooney et al., 2016; Pfeiffer et al., 2018; Kim and Baek, 2019). Conversely, the decreased MAPK signaling in *Tbl1xr1* KO cells may explain the tendency of these cells to differentiate, mimicking a condition of mitogen withdrawal in NSCs *in vitro* (Chojnacki and Weiss, 2008), explaining the microcephaly in both our model and human patients mutated in *TBL1XR1*. Notably, MAPK activity has also been associated with neurite outgrowth and, accordingly, *Tbl1xr1* KO neurons displayed a poorly developed dendritic tree.

We suggest that the F10L substitution in TBL1XR1 is the only mutation, among those studied here, to be similar to the TBL1XR1 loss-of-function. This is probably due to a decreased ability of TBL1XR1^*F*10L^ to interact with the NCOR complex (Nishi et al., 2017), leading to an aberrant MAPK regulation. This mutation was found as *de novo* in Japanese patients affected by sporadic cases of schizophrenia. Interestingly, albeit schizophrenia is a complex and heterogeneous disorder, analyses of expression variance within patients have associated this condition with the MAPK pathway (Igolkina et al., 2018). Moreover, several components of the cascade have been implicated in enhanced risk of SCZ (Xu et al., 2008).

In conclusion, we report for the first time both animal and cellular models to investigate the wide spectrum of neurological manifestations in TBL1XR1-associated disorders. We found behavioral, functional, and developmental impairments that are associated with deregulation of MAPK signaling in *Tbl1xr1* mutant mice. While further analyses will help clarify the exact impact of TBL1XR1 on adult brain functionality, our data shed new light on the molecular pathways at the crossroad of nuclear receptor activity, fundamental cellular and developmental programs, and neurological aspects of TBL1XR1-related human disorders.

## Data Availability Statement

The datasets presented in this study can be found in online repositories. The names of the repository/repositories and accession number(s) can be found in the article/Supplementary Material.

## Ethics Statement

The animal study was reviewed and approved by the OSR Institutional Animal Care and Use Committee, Ministerial Authorization IACUC#820.

## Author Contributions

GM, MZ, MM, FB, and GF performed and analyzed the experiments. LM and EB performed bioinformatic analyses. MI performed behavioral tests. AB helped in the data analysis and in manuscript preparation. ST supervised and performed, with NM, the electrophysiological analyses. MR and SH generated the *Tbl1xr1* flox mouse line. AS supervised the project, conceived, and designed the experiments, analyzed the data, provided with funding, and wrote the manuscript with input from the co-authors. All authors contributed to the article and approved the submitted version.

## Conflict of Interest

The authors declare that the research was conducted in the absence of any commercial or financial relationships that could be construed as a potential conflict of interest.

## Supplementary Material

Supplementary Figure 1 Behavioral deficit of Tbl1xr1 mutant mouse. **(A)** Open field arena to test spontaneous activity as total distance traveled (shown as mean + s.e.m. with dots representing individual samples, examples of traces on the left), n (adult male mice): Ctrl = 21, KO = 17: *p* = 0.1392; statistically compared using Mann-Whitney test. **(B)** Through catwalk assay we measured the duration (left) and the speed (right) of the run (shown as mean + s.e.m.), *n* (adult male mice): Ctrl = 21, KO = 17: duration *p* = 0.1514, statistically compared using *t*-test; speed *p* = 0.1386, statistically compared using *t*-test. **(C)** Thigmotaxis, *n* (adult male mice): Ctrl = 21, KO = 17: *p* = 0.7853; statistically compared using Mann-Whitney test. **(D)** Time in the open arms during elevated plus maze test (shown as mean + s.e.m.), *n* (adult male mice): Ctrl = 10, KO = 10: *p* = 0.2395; statistically compared using Mann-Whitney test. **(E)** Working memory errors (re-entries to arms where the pellet has already been consumed) during the eight-arm radial maze test (shown as means ± SEMs in each experimental day, 1 trial/day), *n* (adult male mice): Ctrl = 21, KO = 17: Multiple comparisons: day 1: ^∗∗∗^*p* < 0.0001; day 2: *p* = 0.1408; day 3: ^****^*p* < 0.0001; day 4: ^∗∗∗^*p* = 0.0007; day 5: *p* > 0.9999; day 6: *p* = 0.1250; day 7: *p* > 0.9999; day 8: *p* > 0.9999; day 9: *p* > 0.9999; day 10: *p* = 0.9367; statistically compared using 2-way ANOVA and Bonferroni’s *post hoc* test. **(F)** Aggressiveness of adult animals measured as the time spent to fight or bite in the resident/intruder test (shown at right as means ± SEMs, with dots representing individual samples), *n*: Ctrl = 20, KO = 19: *p* = 0.1842; statistically compared using unpaired *t*-test. **(G)** Three-chamber test quantified as the time that mouse used to stay in both the different chambers and the sections of chamber A, as well as the time the tested animals spent to sniff both cages (shown as means ± SEMs, with dots representing individual samples), *n* (adult male mice): Ctrl = 21, KO = 17: chamber preference: **(A)** *p* = 0.4776, **(B)** *p* = 0.9723, **(C)** *p* = 0.1780, two-way ANOVA and Sidak’s *post hoc* test; chamber A preference: A1 *p* = 0.2870, A2 *p* = 0.2230, two-way ANOVA and Sidak’s *post hoc* test; sniffing: *p* = 0.3869, unpaired *t*-test.

Supplementary Figure 2 Defects of *Tbl1xr1* mutant neurons. **(A)** Example images and quantification of immunohistochemistry for CTIP2 (left), CUX1 (middle) and SATB2 (right) in cerebral cortex of adult mice (>2 months of age) (shown as mean + s.e.m. with dots representing individual samples): CTIP2: *n*: Ctrl = 10, KO = 10 *p* = 0.1135; CUX1: *n*: Ctrl = 10, KO = 10 ^∗∗∗^*p* = 0.0002; SATB2: *n*: Ctrl = 9, KO = 9 ^∗∗∗^*p* = 0.0001; statistically compared using unpaired *t*-test. **(B)** Spine quantification of DIV 15 hippocampal neurons infected with GFP lentivirus at DIV0 (shown as means ± SEMs, with dots representing individual samples), *n* (replicates): Ctrl = 7, KO = 7: ^****^*p* < 0.0001, unpaired *t*-test. **(C)** Spine quantification of DIV 21 hippocampal neurons infected with GFP lentivirus at DIV0 (shown as means ± SEMs, with dots representing individual samples), *n* (replicates): Ctrl = 9, KO = 9: *p* = 0.6245, unpaired *t*-test. **(D)** Golgi-Cox staining of brains of control and mutant adult mice (>2 months of age). Top, magnification of analyzed dendrite portions. Bottom, quantification of spine density (shown as means ± SEMs, with dots representing individual samples): *n*: Ctrl = 14 (from 2 mice), KO = 14 mice (from 2 mice), *p* = 0.4076; unpaired *t*-test. Scale bars: **(A)** 100 μm; **(B–D)** 10 μm.

Supplementary Figure 3 Defective neural stem cells in *Tbl1xr1* mutant embryos. **(A)** Left, DAPI staining of coronal section of both control and mutant embryonic forebrains (at the stage of E14.5). Right quantification of the cortical thickness in medial (green square) and lateral (red) position (shown as means ± SEMs, with dots representing individual samples), *n* (different animals): Ctrl = 6, KO = 6: medial, ^∗∗^*p* = 0.0014, unpaired *t*-test; lateral *p* = 0.3129, unpaired *t*-test. **(B)** Left, immunohistochemistry for PAX6 (red) and SATB2 (green) on coronal section of E14.5 mouse cortices. Right, quantification (shown as means ± SEMs, with dots representing individual samples), *n* (different animals): Ctrl = 8, KO = 8: PAX6: ^****^*p* < 0.0001; SATB2: ^****^*p* < 0.0001; statistically compared unpaired *t*-test. **(C)** Left, immunocytochemistry of both Ctrl and *Tbl1xr1* KO proliferating *in vitro* NSCs for KI67 counterstained with DAPI. Right (shown as means ± SEMs, with dots representing individual samples), *n* (biological replicates): Ctrl = 8, KO = 8: ^****^*p* < 0.0001, unpaired *t*-test. **(D)** Immunochemistry of both Ctrl and *Tbl1xr1* KO dentate gyrus for GFAP (white), DCX (red), NEUN (green), and KI67 (red) counterstained with DAPI (blue). Below, quantification (shown as means ± SEMs, with dots representing individual samples), n: (different animals) Ctrl = 3, KO = 3: GFAP: *p* = 0.4436; DCX: *p* = 4346; NEUN: *p* = 0.5593; KI67: *p* = 2,810; statistically compared unpaired *t*-test. Scale bars: **(A,C)** 50 μm; **(B)** 25 μm; **(D)** 100 μm.

Supplementary Figure 4 Additional molecular defects of *Tbl1xr1* KO neural stem cells. **(A)** Western blot analysis of whole protein lysates from control and *Tbl1xr1* KO NSCs for the following proteins: NCOR1 and HDAC3 (H3 was used for normalization). On the right, the quantification of the blots for NCOR1 and HDAC3 (shown as mean + s.e.m. with dots representing individual samples): NCOR1: n (biological replicates): Ctrl = 4, KO = 5: ^∗∗^*p* = 0.0047; HDAC3: *n*: Ctrl = 4, KO = 4: ^∗∗^*p* = 0.0020. Statistically compared using unpaired *t*-test. **(B)** Western blot analysis of whole protein lysates from control and *Tbl1xr1* KO adult brains for the following proteins: NCOR1 and HDAC3 (H3 was used for normalization). On the right, the quantification of the blots for NCOR1 and HDAC3 (shown as mean + s.e.m. with dots representing individual samples): NCOR1: *n* (biological replicates): Ctrl = 4, KO = 4: ^∗∗^*p* = 0.0019; HDAC3: *n* (biological replicates): Ctrl = 5, KO = 5: ^∗∗∗^*p* = 0.0006. Statistically compared using unpaired *t*-test. **(C)** Heat map showing genes associated with Wnt pathway according to KEGG. **(D)** Quantification of the immunocytochemistry for PH3 in both Ctrl and *Tbl1xr1* KO proliferating NSCs in a medium either with or without LiCl in the medium (40 mM), counterstained with DAPI. *n* (biological replicates): Ctrl = 6, KO = 6: no LiCl: ^****^*p* < 0.0001; 40 mM LiCl: *p* = 0.9982; one-way ANOVA. **(E)** Quantification of the immunocytochemistry for PH3 in both Ctrl and *Tbl1xr1* KO proliferating NSCs in a medium either with or without IWR1 in the medium (10 mM), counterstained with DAPI. *n*: Ctrl = 6, KO = 6: no IWR1: ^****^*p* < 0.0001; 10 μM IWR1: **^####^***p* < 0.0001; one-way ANOVA. **(F)** Validation of genes related with MAPK deregulated in mutant NSCs by RT-qPCR. Quantification (shown as mean + s.e.m. with dots representing individual samples): *Fgfr1* ^∗∗∗^*p* = 0.0002, *Egfr* ^∗∗^*p* = 0.0012, *Map3k1* ^****^*p* < 0.0001, *Map3k2* ^∗∗∗^*p* = 0.0001, *Map2k2* ^∗∗∗^*p* = 0.0002, *Map2k3* ^∗∗^*p* = 0.0015, *Mapk3* ^****^*p* < 0.0001, *Mapk4* ^∗∗^*p* = 0.0015. Statistically compared using unpaired *t*-test. **(G)** Heat map showing genes associated with MAPK1 as shown in Figure 4F. **(H)** Venn diagram showing the overlap between a curated list of MAPK related genes (black), publicly available ChIP-seq data for NCOR1 binding in IPSCs (GSE1817009, yellow) and publicly available ChIP-seq data for NCOR2 (a.k.a. SMRT) binding in IPSCs (GSE1817010, orange). **(I)** IGV snapshot for ChIP tracks of NCOR1 (GSE1817009, yellow) and NCOR2 (a.k.a. SMRT) (GSE1817010, orange) showing peaks near the *Map2k2* gene (in IPSC). **(J)** Scatter plot showing Homer results for motif enrichment analysis in promoters (–1,000 + 100 bp from TSS) of the downregulated genes in *Tbl1xr1* KO compared to control NSCs. **(K)** Validation of genes encoding for E2F transcription factors deregulated in mutant NSCs by RT-qPCR. Quantification (shown as mean + s.e.m. with dots representing individual samples): *E2f1* ^****^*p* < 0.0001, *E2f2* ^****^*p* < 0.0001. Statistically compared using unpaired *t*-test. See also Supplementary Tables S3, S4).

Supplementary Figure 5 Additional characterization of the complementation of *Tbl1xr1* KO neural stem cells. **(A)** Growth curve of the indicated adherent *in vitro* NSCs, see also Figure 5B. **(B)** Left, histograms showing sphere’s diameters of indicated NSCs cultured as neurospheres at the indicated days *in vitro* after disaggregation. STATS. Right, examples of microphotographs of the neurospheres of the indicated NSCs cultured at the indicated days *in vitro* after disaggregation. **(C)** Immunocytochemistry of the indicated proliferating NSCs for phosphor histone 3 (PH3) counterstained with DAPI. Quantification is shown in Figure 5C. **(D)** Immunocytochemistry of the indicated proliferating NSCs for TUJ1 (red) and S100b (green) counterstained with DAPI (left) and O4 counterstained with DAPI (right). Quantification (shown as mean + s.e.m. with dots representing individual samples): TUJ1: is shown in Figure 5C; S100b: Ctrl + LV Mock vs.: KO + LV Mock ^****^*p* < 0.0001; KO + LV WT *p* = 0.5496; KO + LV F10L ^****^*p* < 0.0001; KO + LV G70D *p* = 0.2906; KO + LV L282P *p* = 0.8682; KO + LV Y446C *p* = 0.5420; one-way ANOVA with Dunnett’s multiple comparisons test. O4: Ctrl + LV Mock vs.: KO + LV Mock ^∗∗∗^*p* = 0.0002; KO + LV WT *p* = 0.3449; KO + LV F10L ^****^*p* < 0.0001; KO + LV G70D *p* = 0.3247; KO + LV L282P *p* = 0.6425; KO + LV Y446C *p* = 0.7304; one-way ANOVA with Dunnett’s multiple comparisons test. Scale bars: **(A)** 60 μm; **(C,D)** 50 μm.

Supplementary Table 1 List of oligonucleotides used as primers for RT-qPCR in this work.

Supplementary Table 2 Sheet 1: Results of DESeq2 analysis of RNAseq data between Tbl1xr1 KO and control (GFP) NSCs. Sheet 2: Lists of differentially expressed genes (DEGs) divided in down and upregulated. Sheet 3: Complete lists of Gene Ontology and KEEG pathway analyses performed on DEGs. Sheet 4: Lists as in Venn diagram in Figure 4E. Sheet 5: GO analysis of genes that are both upregulated and TBL1XR1 targets. Sheet 6: GO analysis of genes that are both downregulated and TBL1XR1 targets.

Supplementary Table 3 List of Wnt signaling related genes (curated using KEGG) as in heatmap in Supplementary Figure S4C.

Supplementary Table 4 Sheet 1: List of MAPK signaling related genes (curated using different databases) used in Venn diagram in Supplementary Figure S4H. Sheet 2: List of MAPK signaling related genes downregulated in Tbl1xr1 NSCs NSCs as in scheme of the Figure 4F. Sheet 3: Lists as in Venn diagram in Supplementary Figure S4H.

Supplementary Table 5 Full list of Motif Enrichment Analysis by Homer using –1,000 + 100 bp from TSS as parameters.

## References

[B1] Azari H. Rahman M. Sharififar S. Reynolds B. A. (2010). Isolation and expansion of the adult mouse neural stem cells using the neurosphere assay. *J. Vis. Exp.* 45:2393. 10.3791/2393 21113123 PMC3159587

[B2] Bem J. Brożko N. Chakraborty C. Lipiec M. A. Koziński K. Nagalski A. (2019). Wnt/β-catenin signaling in brain development and mental disorders: keeping TCF7L2 in mind. *FEBS Lett.* 593 1654–1674. 10.1002/1873-3468.13502 31218672 PMC6772062

[B3] Borrell V. (2019). Recent advances in understanding neocortical development. *F1000Res* 8:F1000FacultyRev–1791. 10.12688/f1000research.20332.1 31681469 PMC6816450

[B4] Castelo-Branco G. Lilja T. Wallenborg K. Falcao A. M. Marques S. C. Gracias A. (2014). Neural stem cell differentiation is dictated by distinct actions of nuclear receptor corepressors and histone deacetylases. *Stem Cell Reports* 3 502–515. 10.1016/j.stemcr.2014.07.008 25241747 PMC4266002

[B5] Chenn A. Walsh C. A. (2002). Regulation of cerebral cortical size by control of cell cycle exit in neural precursors. *Science* 297 365–369. 10.1126/science.1074192 12130776

[B6] Choi H. K. Choi K. C. Yoo J. Y. Song M. Ko S. J. Kim C. H. (2011). Reversible SUMOylation of TBL1-TBLR1 regulates beta-catenin-mediated Wnt signaling. *Mol. Cell* 43 203–216. 10.1016/j.molcel.2011.05.027 21777810

[B7] Chojnacki A. Weiss S. (2008). Production of neurons, astrocytes and oligodendrocytes from mammalian CNS stem cells. *Nat. Protoc.* 3 935–940. 10.1038/nprot.2008.55 18536641

[B8] Clément-Lacroix P. Ai M. Morvan F. Roman-Roman S. Vayssière B. Belleville C. (2005). Lrp5-independent activation of Wnt signaling by lithium chloride increases bone formation and bone mass in mice. *Proc. Natl. Acad. Sci. U.S.A.* 102 17406–17411. 10.1073/pnas.0505259102 16293698 PMC1297659

[B9] Colombo E. Giannelli S. G. Galli R. Tagliafico E. Foroni C. Tenedini E. (2006). Embryonic stem-derived versus somatic neural stem cells: a comparative analysis of their developmental potential and molecular phenotype. *Stem Cells* 24 825–834. 10.1634/stemcells.2005-0313 16339994

[B10] Ebert D. H. Gabel H. W. Robinson N. D. Kastan N. R. Hu L. S. Cohen S. (2013). Activity-dependent phosphorylation of MeCP2 threonine 308 regulates interaction with NCoR. *Nature* 499 341–345. 10.1038/nature12348 23770587 PMC3922283

[B11] Ferreira T. A. Blackman A. V. Oyrer J. Jayabal S. Chung A. J. Watt A. J. (2014). Neuronal morphometry directly from bitmap images. *Nat. Methods* 11 982–984. 10.1038/nmeth.3125 25264773 PMC5271921

[B12] Galli R. Gritti A. Bonfanti L. Vescovi A. L. (2003). Neural stem cells: an overview. *Circ. Res.* 92 598–608. 10.1161/01.RES.0000065580.02404.F4 12676811

[B13] Heinen C. A. Jongejan A. Watson P. J. Redeker B. Boelen A. Boudzovitch-Surovtseva O. (2016). A specific mutation in TBL1XR1 causes Pierpont syndrome. *J. Med. Genet.* 53 330–337. 10.1136/jmedgenet-2015-103233 26769062 PMC4853543

[B14] Heldring N. Nyman U. Lönnerberg P. Onnestam S. Herland A. Holmberg J. (2014). NCoR controls glioblastoma tumor cell characteristics. *Neuro Oncol.* 16 241–249. 10.1093/neuonc/not214 24335696 PMC3895387

[B15] Hermanson O. Jepsen K. Rosenfeld M. G. (2002). N-CoR controls differentiation of neural stem cells into astrocytes. *Nature* 419 934–939. 10.1038/nature01156 12410313

[B16] Hirabayashi Y. Itoh Y. Tabata H. Nakajima K. Akiyama T. Masuyama N. (2004). The Wnt/beta-catenin pathway directs neuronal differentiation of cortical neural precursor cells. *Development* 131 2791–2801. 10.1242/dev.01165 15142975

[B17] Holowacz T. Huelsken J. Dufort D. van der Kooy D. (2011). Neural stem cells are increased after loss of β-catenin, but neural progenitors undergo cell death. *Eur. J. Neurosci.* 33 1366–1375. 10.1111/j.1460-9568.2011.07632.x 21375603

[B18] Huang D. W. Sherman B. T. Lempicki R. A. (2009). Bioinformatics enrichment tools: paths toward the comprehensive functional analysis of large gene lists. *Nucleic Acids Res.* 37 1–13. 10.1093/nar/gkn923 19033363 PMC2615629

[B19] Iemolo A. Montilla-Perez P. Lai I.-C. Meng Y. Nolan S. Wen J. (2020). A cell type-specific expression map of NCoR1 and SMRT transcriptional co-repressors in the mouse brain. *J. Comp. Neurol.* 528 2218–2238. 10.1002/cne.24886 32072640 PMC7368833

[B20] Igolkina A. A. Armoskus C. Newman J. R. B. Evgrafov O. V. McIntyre L. M. Nuzhdin S. V. (2018). Analysis of gene expression variance in schizophrenia using structural equation modeling. *Front. Mol. Neurosci.* 11:192. 10.3389/fnmol.2018.00192 29942251 PMC6004421

[B21] Ishizuka T. Lazar M. A. (2005). The nuclear receptor corepressor deacetylase activating domain is essential for repression by thyroid hormone receptor. *Mol. Endocrinol.* 19 1443–1451. 10.1210/me.2005-0009 15695367

[B22] Iwama K. Mizuguchi T. Takeshita E. Nakagawa E. Okazaki T. Nomura Y. (2019). Genetic landscape of Rett syndrome-like phenotypes revealed by whole exome sequencing. *J. Med. Genet.* 56 396–407. 10.1136/jmedgenet-2018-105775 30842224

[B23] Jepsen K. Hermanson O. Onami T. M. Gleiberman A. S. Lunyak V. McEvilly R. J. (2000). Combinatorial roles of the nuclear receptor corepressor in transcription and development. *Cell* 102 753–763. 10.1016/s0092-8674(00)00064-7 11030619

[B24] Jepsen K. Solum D. Zhou T. McEvilly R. J. Kim H. J. Glass C. K. (2007). SMRT-mediated repression of an H3K27 demethylase in progression from neural stem cell to neuron. *Nature* 450 415–419. 10.1038/nature06270 17928865

[B25] Jimenez-Pascual A. Siebzehnrubl F. A. (2019). Fibroblast growth factor receptor functions in glioblastoma. *Cells* 8:715. 10.3390/cells8070715 31337028 PMC6678715

[B26] Kim Y. E. Baek S. T. (2019). Neurodevelopmental aspects of RASopathies. *Mol. Cells* 42 441–447. 10.14348/molcells.2019.0037 31250618 PMC6602148

[B27] Kim D. Pertea G. Trapnell C. Pimentel H. Kelley R. Salzberg S. L. (2013). TopHat2: accurate alignment of transcriptomes in the presence of insertions, deletions and gene fusions. *Genome Biol.* 14 R36. 10.1186/gb-2013-14-4-r36 PMC4053844 23618408

[B28] Kong Y. Zhou W. Sun Z. (2020). Nuclear receptor corepressors in intellectual disability and autism. *Mol. Psychiatry* 25 2220–2236. 10.1038/s41380-020-0667-y 32034290 PMC7842082

[B29] Kruusvee V. Lyst M. J. Taylor C. Tarnauskaitė Ž Bird A. P. Cook A. G. (2017). Structure of the MeCP2-TBLR1 complex reveals a molecular basis for Rett syndrome and related disorders. *Proc. Natl. Acad. Sci. U.S.A.* 114 E3243–E3250. 10.1073/pnas.1700731114 28348241 PMC5402415

[B30] Kuwabara T. Hsieh J. Muotri A. Yeo G. Warashina M. Lie D. C. (2009). Wnt-mediated activation of NeuroD1 and retro-elements during adult neurogenesis. *Nat. Neurosci.* 12 1097–1105. 10.1038/nn.2360 19701198 PMC2764260

[B31] Laskowski R. A. Tyagi N. Johnson D. Joss S. Kinning E. McWilliam C. (2016). Integrating population variation and protein structural analysis to improve clinical interpretation of missense variation: application to the WD40 domain. *Hum. Mol. Genet.* 25 927–935. 10.1093/hmg/ddv625 26740553 PMC4754046

[B32] Leo L. M. Almeida-Corrêa S. Canetti C. A. Amaral O. B. Bozza F. A. Pamplona F. A. (2014). Age-dependent relevance of endogenous 5-lipoxygenase derivatives in anxiety-like behavior in mice. *PLoS One* 9:e85009. 10.1371/journal.pone.0085009 24416334 PMC3885659

[B33] Li J. Wang C. Y. (2008). TBL1-TBLR1 and beta-catenin recruit each other to Wnt target-gene promoter for transcription activation and oncogenesis. *Nat. Cell Biol.* 10 160–169. 10.1038/ncb1684 18193033

[B34] Li J. Y. Daniels G. Wang J. Zhang X. (2015). TBL1XR1 in physiological and pathological states. *Am. J. Clin. Exp. Urol.* 3 13–23. 26069883 PMC4446378

[B35] Love M. I. Huber W. Anders S. (2014). Moderated estimation of fold change and dispersion for RNA-seq data with DESeq2. *Genome Biol.* 15:550. 10.1186/s13059-014-0550-8 25516281 PMC4302049

[B36] Lyst M. J. Ekiert R. Ebert D. H. Merusi C. Nowak J. Selfridge J. (2013). Rett syndrome mutations abolish the interaction of MeCP2 with the NCoR/SMRT co-repressor. *Nat. Neurosci.* 16 898–902. 10.1038/nn.3434 23770565 PMC3786392

[B37] MacDonald B. T. Tamai K. He X. (2009). Wnt/beta-catenin signaling: components, mechanisms, and diseases. *Dev. Cell* 17 9–26. 10.1016/j.devcel.2009.06.016 19619488 PMC2861485

[B38] Marinaro C. Pannese M. Weinandy F. Sessa A. Bergamaschi A. Taketo M. M. (2012). Wnt signaling has opposing roles in the developing and the adult brain that are modulated by Hipk1. *Cereb. Cortex* 22 2415–2427. 10.1093/cercor/bhr320 22095214

[B39] Martins-Neves S. R. Paiva-Oliveira D. I. Fontes-Ribeiro C. Bovée J. V. M. G. Cleton-Jansen A. M. Gomes C. M. F. (2018). IWR-1, a tankyrase inhibitor, attenuates Wnt/β-catenin signaling in cancer stem-like cells and inhibits in vivo the growth of a subcutaneous human osteosarcoma xenograft. *Cancer Lett.* 414 1–15. 10.1016/j.canlet.2017.11.004 29126913

[B40] Mossahebi-Mohammadi M. Quan M. Zhang J. S. Li X. (2020). FGF signaling pathway: a Key regulator of stem cell pluripotency. *Front. Cell Dev. Biol.* 8:79. 10.3389/fcell.2020.00079 32133359 PMC7040165

[B41] Nikolai B. C. Lanz R. B. York B. Dasgupta S. Mitsiades N. Creighton C. J. (2016). HER2 signaling drives DNA anabolism and proliferation through SRC-3 phosphorylation and E2F1-regulated genes. *Cancer Res.* 76 1463–1475. 10.1158/0008-5472.CAN-15-2383 26833126 PMC4794399

[B42] Nishi A. Numata S. Tajima A. Zhu X. Ito K. Saito A. (2017). De novo non-synonymous TBL1XR1 mutation alters Wnt signaling activity. *Sci. Rep.* 7:2887. 10.1038/s41598-017-02792-z 28588275 PMC5460159

[B43] O’Roak B. J. Vives L. Fu W. Egertson J. D. Stanaway I. B. Phelps I. G. (2012). Multiplex targeted sequencing identifies recurrently mutated genes in autism spectrum disorders. *Science* 338 1619–1622. 10.1126/science.1227764 23160955 PMC3528801

[B44] Ohkubo Y. Uchida A. O. Shin D. Partanen J. Vaccarino F. M. (2004). Fibroblast growth factor receptor 1 is required for the proliferation of hippocampal progenitor cells and for hippocampal growth in mouse. *J. Neurosci.* 24 6057–6069. 10.1523/JNEUROSCI.1140-04.2004 15240797 PMC6729672

[B45] Oliva C. A. Montecinos-Oliva C. Inestrosa N. C. (2018). Wnt signaling in the central nervous system: new insights in health and disease. *Prog. Mol. Biol. Transl. Sci.* 153 81–130. 10.1016/bs.pmbts.2017.11.018 29389523

[B46] Papale A. d’Isa R. Menna E. Cerovic M. Solari N. Hardingham N. (2017). Severe intellectual disability and enhanced gamma-aminobutyric acidergic synaptogenesis in a novel model of rare RASopathies. *Biol. Psychiatry* 81 179–192. 10.1016/j.biopsych.2016.06.016 27587266

[B47] Perissi V. Aggarwal A. Glass C. K. Rose D. W. Rosenfeld M. G. (2004). A corepressor/coactivator exchange complex required for transcriptional activation by nuclear receptors and other regulated transcription factors. *Cell* 116 511–526. 10.1016/s0092-8674(04)00133-3 14980219

[B48] Perissi V. Scafoglio C. Zhang J. Ohgi K. A. Rose D. W. Glass C. K. (2008). TBL1 and TBLR1 phosphorylation on regulated gene promoters overcomes dual CtBP and NCoR/SMRT transcriptional repression checkpoints. *Mol. Cell* 29 755–766. 10.1016/j.molcel.2008.01.020 18374649 PMC2364611

[B49] Perissi V. Jepsen K. Glass C. K. Rosenfeld M. G. (2010). Deconstructing repression: evolving models of co-repressor action. *Nat. Rev. Genet.* 11 109–123. 10.1038/nrg2736 20084085

[B50] Pfeiffer V. Götz R. Camarero G. Heinsen H. Blum R. Rapp U. R. (2018). Impaired neuronal maturation of hippocampal neural progenitor cells in mice lacking CRAF. *PLoS One* 13:e0192067. 10.1371/journal.pone.0192067 29590115 PMC5873938

[B51] Pons L. Cordier M. P. Labalme A. Till M. Louvrier C. Schluth-Bolard C. (2015). A new syndrome of intellectual disability with dysmorphism due to TBL1XR1 deletion. *Am. J. Med. Genet. A.* 167A 164–168. 10.1002/ajmg.a.36759 25425123

[B52] Rauen K. A. (2013). The RASopathies. *Annu. Rev. Genomics Hum. Genet.* 14 355–369. 10.1146/annurev-genom-091212-153523 23875798 PMC4115674

[B53] Riehmer V. Erger F. Herkenrath P. Seland S. Jackels M. Wiater A. (2017). A heritable microduplication encompassing TBL1XR1 causes a genomic sister-disorder for the 3q26.32 microdeletion syndrome. *Am. J. Med. Genet. A.* 173 2132–2138. 10.1002/ajmg.a.38285 28574232

[B54] Rohm M. Sommerfeld A. Strzoda D. Jones A. Sijmonsma T. P. Rudofsky G. (2013). Transcriptional cofactor TBLR1 controls lipid mobilization in white adipose tissue. *Cell Metab.* 17 575–585. 10.1016/j.cmet.2013.02.010 23499424

[B55] Rooney G. E. Goodwin A. F. Depeille P. Sharir A. Schofield C. M. Yeh E. (2016). Human iPS cell-derived neurons uncover the impact of increased Ras signaling in costello syndrome. *J. Neurosci.* 36 142–152. 10.1523/JNEUROSCI.1547-15.2016 26740656 PMC4701956

[B56] Saitsu H. Tohyama J. Walsh T. Kato M. Kobayashi Y. Lee M. (2014). A girl with West syndrome and autistic features harboring a de novo TBL1XR1 mutation. *J. Hum. Genet.* 59 581–583. 10.1038/jhg.2014.71 25102098

[B57] Sajan S. A. Jhangiani S. N. Muzny D. M. Gibbs R. A. Lupski J. R. Glaze D. G. (2017). Enrichment of mutations in chromatin regulators in people with Rett syndrome lacking mutations in MECP2. *Genet. Med.* 19 13–19. 10.1038/gim.2016.42 27171548 PMC5107176

[B58] Sakaguchi Y. Uehara T. Suzuki H. Sakamoto Y. Fujiwara M. Kosaki K. (2018). Haploinsufficiency of NCOR1 associated with autism spectrum disorder, scoliosis, and abnormal palatogenesis. *Am. J. Med. Genet. A.* 176 2466–2469. 10.1002/ajmg.a.40354 30289594

[B59] Silverman J. L. Yang M. Lord C. Crawley J. N. (2010). Behavioural phenotyping assays for mouse models of autism. *Nat. Rev. Neurosci.* 11 490–502. 10.1038/nrn2851 20559336 PMC3087436

[B60] Sessa A. Fagnocchi L. Mastrototaro G. Massimino L. Zaghi M. Indrigo M. (2019). SETD5 Regulates Chromatin Methylation State and Preserves Global Transcriptional Fidelity during Brain Development and Neuronal Wiring. *Neuron*, 104 271–289. 10.1016/j.neuron.2019.07.013 31515109

[B61] Stessman H. A. Xiong B. Coe B. P. Wang T. Hoekzema K. Fenckova M. (2017). Targeted sequencing identifies 91 neurodevelopmental-disorder risk genes with autism and developmental-disability biases. *Nat. Genet.* 49 515–526. 10.1038/ng.3792 28191889 PMC5374041

[B62] Sun Z. Xu Y. (2020). Nuclear Receptor Coactivators (NCOAs) and Corepressors (NCORs) in the Brain. *Endocrinology* 161 1–12. 10.1210/endocr/bqaa083 32449767 PMC7351129

[B63] Thapar A. Cooper M. Rutter M. (2017). Neurodevelopmental disorders. *Lancet Psychiatry* 4 339–346. 10.1016/S2215-0366(16)30376-5 27979720

[B64] Vaqueiro A. C. de Oliveira C. P. Cordoba M. S. Versiani B. R. de Carvalho C. X. Alves Rodrigues P. G. (2018). Expanding the spectrum of TBL1XR1 deletion: report of a patient with brain and cardiac malformations. *Eur. J. Med. Genet.* 61 29–33. 10.1016/j.ejmg.2017.10.008 29038029

[B65] Vithayathil J. Pucilowska J. Goodnough L. H. Atit R. P. Landreth G. E. (2015). Dentate Gyrus development requires ERK activity to maintain progenitor population and MAPK pathway feedback regulation. *J. Neurosci.* 35 6836–6848. 10.1523/JNEUROSCI.4196-14.2015 25926459 PMC4412899

[B66] Wang T. Guo H. Xiong B. Stessman H. A. Wu H. Coe B. P. (2016). De novo genic mutations among a Chinese autism spectrum disorder cohort. *Nat Commun.* 7:13316. 10.1038/ncomms13316 27824329 PMC5105161

[B67] Yoon H. G. Chan D. W. Huang Z. Q. Li J. Fondell J. D. Qin J. (2003). Purification and functional characterization of the human N-CoR complex: the roles of HDAC3, TBL1 and TBLR1. *EMBO J.* 22 1336–1346. 10.1093/emboj/cdg120 12628926 PMC151047

[B68] Yoon H. G. Choi Y. Cole P. A. Wong J. (2005). Reading and function of a histone code involved in targeting corepressor complexes for repression. *Mol. Cell Biol.* 25 324–335. 10.1128/MCB.25.1.324-335.2005 15601853 PMC538779

[B69] Xu B. Roos J. L. Levy S. van Rensburg E. J. Gogos J. A. Karayiorgou M. (2008). Strong association of de novo copy number mutations with sporadic schizophrenia. *Nat. Genet.* 40 880–885. 10.1038/ng.162 18511947

[B70] Zhou W. He Y. Rehman A. U. Kong Y. Hong S. Ding G. (2019). Loss of function of NCOR1 and NCOR2 impairs memory through a novel GABAergic hypothalamus-CA3 projection. *Nat. Neurosci.* 22 205–217. 10.1038/s41593-018-0311-1 30664766 PMC6361549

[B71] Zhuang Q. Li W. Benda C. Huang Z. Ahmed T. Liu P. (2018). NCoR/SMRT co-repressors cooperate with c-MYC to create an epigenetic barrier to somatic cell reprogramming. *Nat. Cell Biol.* 20 400–412. 10.1038/s41556-018-0047-x 29531310

