# Supplementary figures and images for: TBL1XR1 Ensures Balanced Neural Development Through NCOR Complex-Mediated Regulation of the MAPK Pathway
Source: Front Cell Dev Biol. 2021 Feb 23;9:641410. doi: 10.3389/fcell.2021.641410 (PMC7940385; doi:10.3389/fcell.2021.641410)

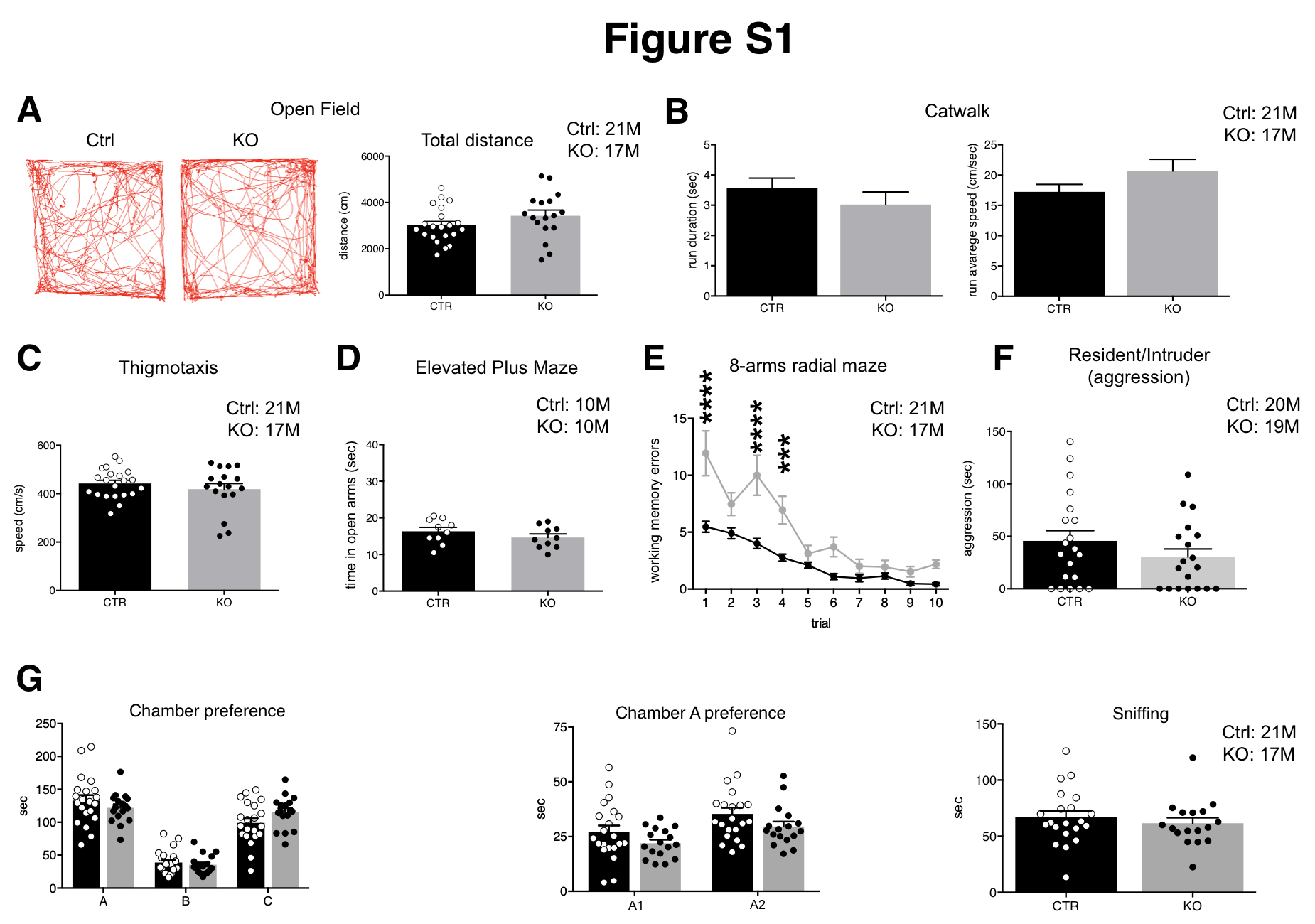

Supplement: Supplementary Figure 1 — Behavioral deficit of Tbl1xr1 mutant mouse. (A) Open field arena to test spontaneous activity as total distance traveled (shown as mean + s.e.m. with dots representing individual samples, examples of traces on the left), n (adult male mice): Ctrl = 21, KO = 17: p = 0.1392; statistically compared using Mann-Whitney test. (B) Through catwalk assay we measured the duration (left) and the speed (right) of the run (shown as mean + s.e.m.), n (adult male mice): Ctrl = 21, KO = 17: duration p = 0.1514, statistically compared using t-test; speed p = 0.1386, statistically compared using t-test. (C) Thigmotaxis, n (adult male mice): Ctrl = 21, KO = 17: p = 0.7853; statistically compared using Mann-Whitney test. (D) Time in the open arms during elevated plus maze test (shown as mean + s.e.m.), n (adult male mice): Ctrl = 10, KO = 10: p = 0.2395; statistically compared using Mann-Whitney test. (E) Working memory errors (re-entries to arms where the pellet has already been consumed) during the eight-arm radial maze test (shown as means ± SEMs in each experimental day, 1 trial/day), n (adult male mice): Ctrl = 21, KO = 17: Multiple comparisons: day 1: ∗∗∗p < 0.0001; day 2: p = 0.1408; day 3: ****p < 0.0001; day 4: ∗∗∗p = 0.0007; day 5: p > 0.9999; day 6: p = 0.1250; day 7: p > 0.9999; day 8: p > 0.9999; day 9: p > 0.9999; day 10: p = 0.9367; statistically compared using 2-way ANOVA and Bonferroni’s post hoc test. (F) Aggressiveness of adult animals measured as the time spent to fight or bite in the resident/intruder test (shown at right as means ± SEMs, with dots representing individual samples), n: Ctrl = 20, KO = 19: p = 0.1842; statistically compared using unpaired t-test. (G) Three-chamber test quantified as the time that mouse used to stay in both the different chambers and the sections of chamber A, as well as the time the tested animals spent to sniff both cages (shown as means ± SEMs, with dots representing individual samples), n (adult male mice): Ctrl = 21, KO = 17: cha [file Image_1.JPEG]

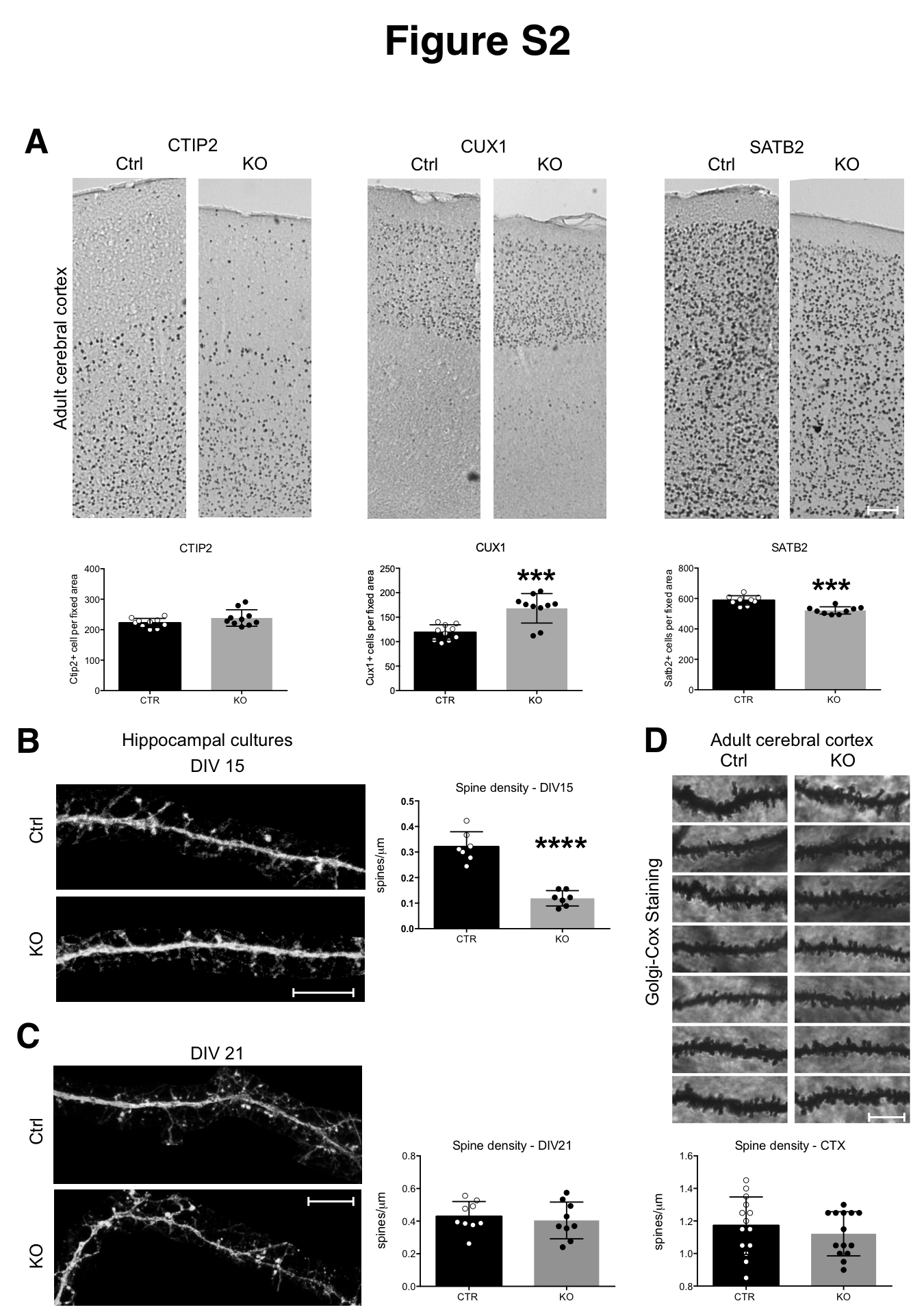

Supplement: Supplementary Figure 2 — Defects of Tbl1xr1 mutant neurons. (A) Example images and quantification of immunohistochemistry for CTIP2 (left), CUX1 (middle) and SATB2 (right) in cerebral cortex of adult mice (>2 months of age) (shown as mean + s.e.m. with dots representing individual samples): CTIP2: n: Ctrl = 10, KO = 10 p = 0.1135; CUX1: n: Ctrl = 10, KO = 10 ∗∗∗p = 0.0002; SATB2: n: Ctrl = 9, KO = 9 ∗∗∗p = 0.0001; statistically compared using unpaired t-test. (B) Spine quantification of DIV 15 hippocampal neurons infected with GFP lentivirus at DIV0 (shown as means ± SEMs, with dots representing individual samples), n (replicates): Ctrl = 7, KO = 7: ****p < 0.0001, unpaired t-test. (C) Spine quantification of DIV 21 hippocampal neurons infected with GFP lentivirus at DIV0 (shown as means ± SEMs, with dots representing individual samples), n (replicates): Ctrl = 9, KO = 9: p = 0.6245, unpaired t-test. (D) Golgi-Cox staining of brains of control and mutant adult mice (>2 months of age). Top, magnification of analyzed dendrite portions. Bottom, quantification of spine density (shown as means ± SEMs, with dots representing individual samples): n: Ctrl = 14 (from 2 mice), KO = 14 mice (from 2 mice), p = 0.4076; unpaired t-test. Scale bars: (A) 100 μm; (B–D) 10 μm. [file Image_2.JPEG]

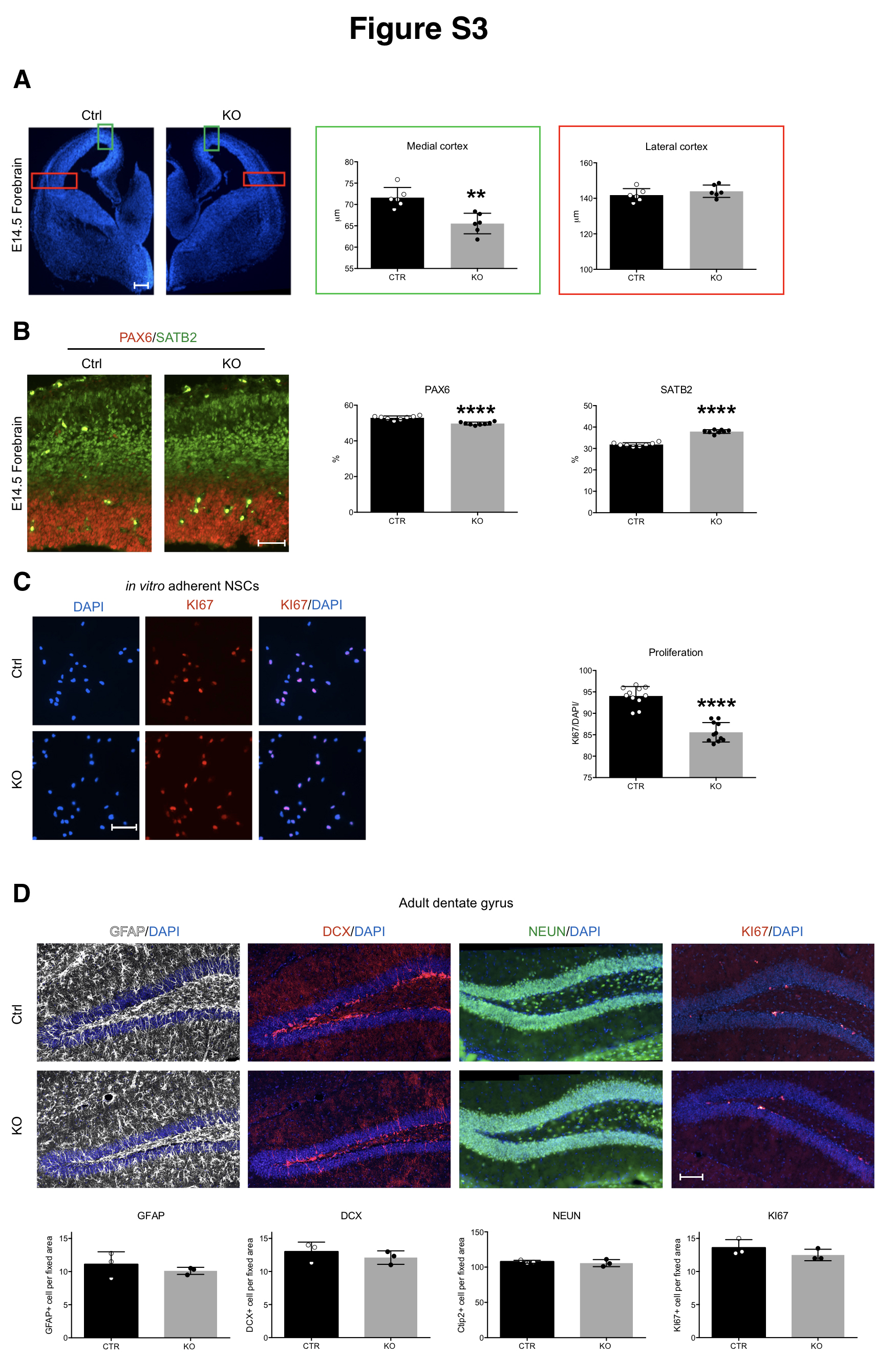

Supplement: Supplementary Figure 3 — Defective neural stem cells in Tbl1xr1 mutant embryos. (A) Left, DAPI staining of coronal section of both control and mutant embryonic forebrains (at the stage of E14.5). Right quantification of the cortical thickness in medial (green square) and lateral (red) position (shown as means ± SEMs, with dots representing individual samples), n (different animals): Ctrl = 6, KO = 6: medial, ∗∗p = 0.0014, unpaired t-test; lateral p = 0.3129, unpaired t-test. (B) Left, immunohistochemistry for PAX6 (red) and SATB2 (green) on coronal section of E14.5 mouse cortices. Right, quantification (shown as means ± SEMs, with dots representing individual samples), n (different animals): Ctrl = 8, KO = 8: PAX6: ****p < 0.0001; SATB2: ****p < 0.0001; statistically compared unpaired t-test. (C) Left, immunocytochemistry of both Ctrl and Tbl1xr1 KO proliferating in vitro NSCs for KI67 counterstained with DAPI. Right (shown as means ± SEMs, with dots representing individual samples), n (biological replicates): Ctrl = 8, KO = 8: ****p < 0.0001, unpaired t-test. (D) Immunochemistry of both Ctrl and Tbl1xr1 KO dentate gyrus for GFAP (white), DCX (red), NEUN (green), and KI67 (red) counterstained with DAPI (blue). Below, quantification (shown as means ± SEMs, with dots representing individual samples), n: (different animals) Ctrl = 3, KO = 3: GFAP: p = 0.4436; DCX: p = 4346; NEUN: p = 0.5593; KI67: p = 2,810; statistically compared unpaired t-test. Scale bars: (A,C) 50 μm; (B) 25 μm; (D) 100 μm. [file Image_3.JPEG]

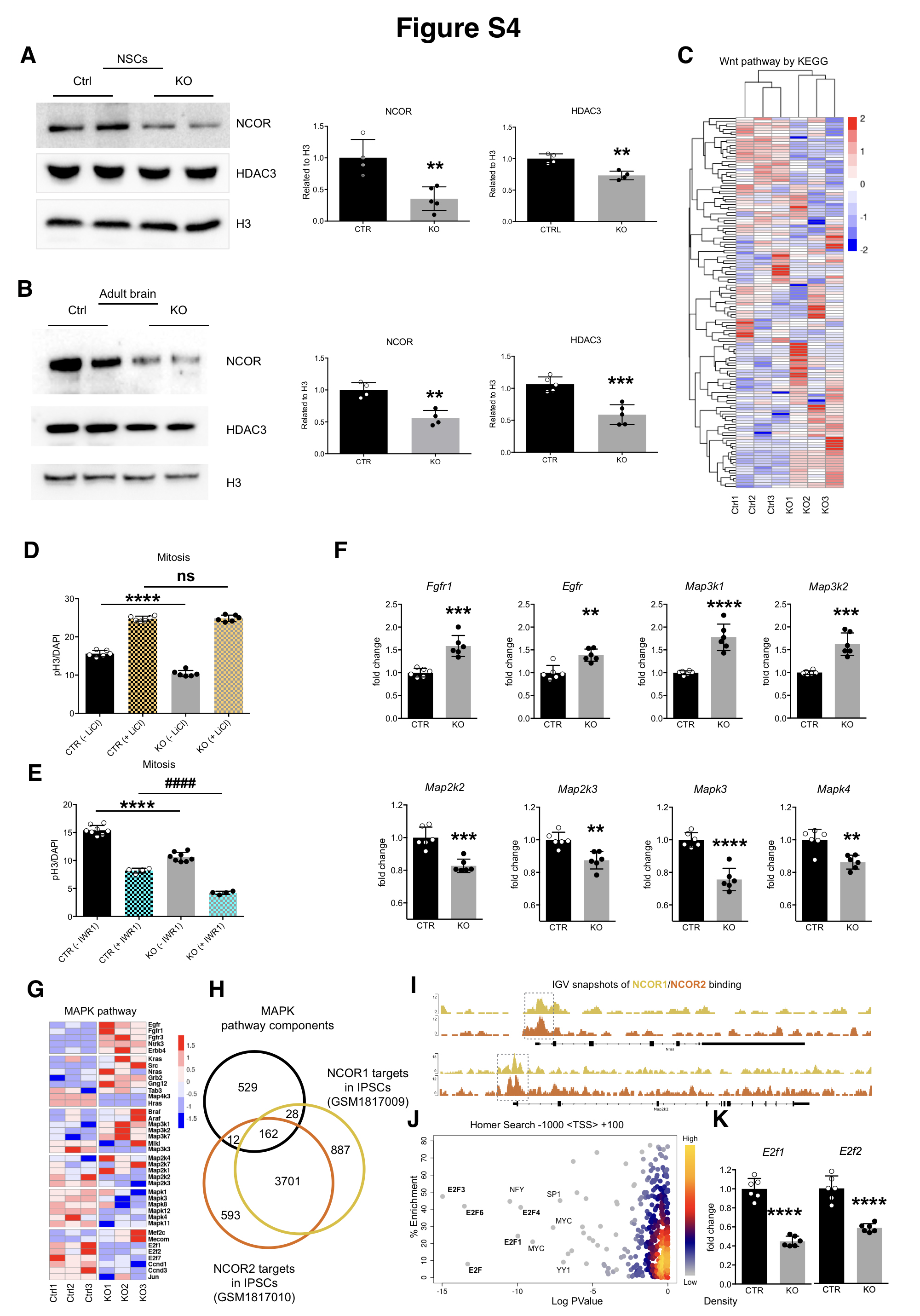

Supplement: Supplementary Figure 4 — Additional molecular defects of Tbl1xr1 KO neural stem cells. (A) Western blot analysis of whole protein lysates from control and Tbl1xr1 KO NSCs for the following proteins: NCOR1 and HDAC3 (H3 was used for normalization). On the right, the quantification of the blots for NCOR1 and HDAC3 (shown as mean + s.e.m. with dots representing individual samples): NCOR1: n (biological replicates): Ctrl = 4, KO = 5: ∗∗p = 0.0047; HDAC3: n: Ctrl = 4, KO = 4: ∗∗p = 0.0020. Statistically compared using unpaired t-test. (B) Western blot analysis of whole protein lysates from control and Tbl1xr1 KO adult brains for the following proteins: NCOR1 and HDAC3 (H3 was used for normalization). On the right, the quantification of the blots for NCOR1 and HDAC3 (shown as mean + s.e.m. with dots representing individual samples): NCOR1: n (biological replicates): Ctrl = 4, KO = 4: ∗∗p = 0.0019; HDAC3: n (biological replicates): Ctrl = 5, KO = 5: ∗∗∗p = 0.0006. Statistically compared using unpaired t-test. (C) Heat map showing genes associated with Wnt pathway according to KEGG. (D) Quantification of the immunocytochemistry for PH3 in both Ctrl and Tbl1xr1 KO proliferating NSCs in a medium either with or without LiCl in the medium (40 mM), counterstained with DAPI. n (biological replicates): Ctrl = 6, KO = 6: no LiCl: ****p < 0.0001; 40 mM LiCl: p = 0.9982; one-way ANOVA. (E) Quantification of the immunocytochemistry for PH3 in both Ctrl and Tbl1xr1 KO proliferating NSCs in a medium either with or without IWR1 in the medium (10 mM), counterstained with DAPI. n: Ctrl = 6, KO = 6: no IWR1: ****p < 0.0001; 10 μM IWR1: ####p < 0.0001; one-way ANOVA. (F) Validation of genes related with MAPK deregulated in mutant NSCs by RT-qPCR. Quantification (shown as mean + s.e.m. with dots representing individual samples): Fgfr1 ∗∗∗p = 0.0002, Egfr ∗∗p = 0.0012, Map3k1 ****p < 0.0001, Map3k2 ∗∗∗p = 0.0001, Map2k2 ∗∗∗p = 0.0002, Map2k3 ∗∗p = 0.0015, Mapk3 ****p < 0.0001, Mapk4 ∗∗p = 0.0015. Statistically compare [file Image_4.JPEG]

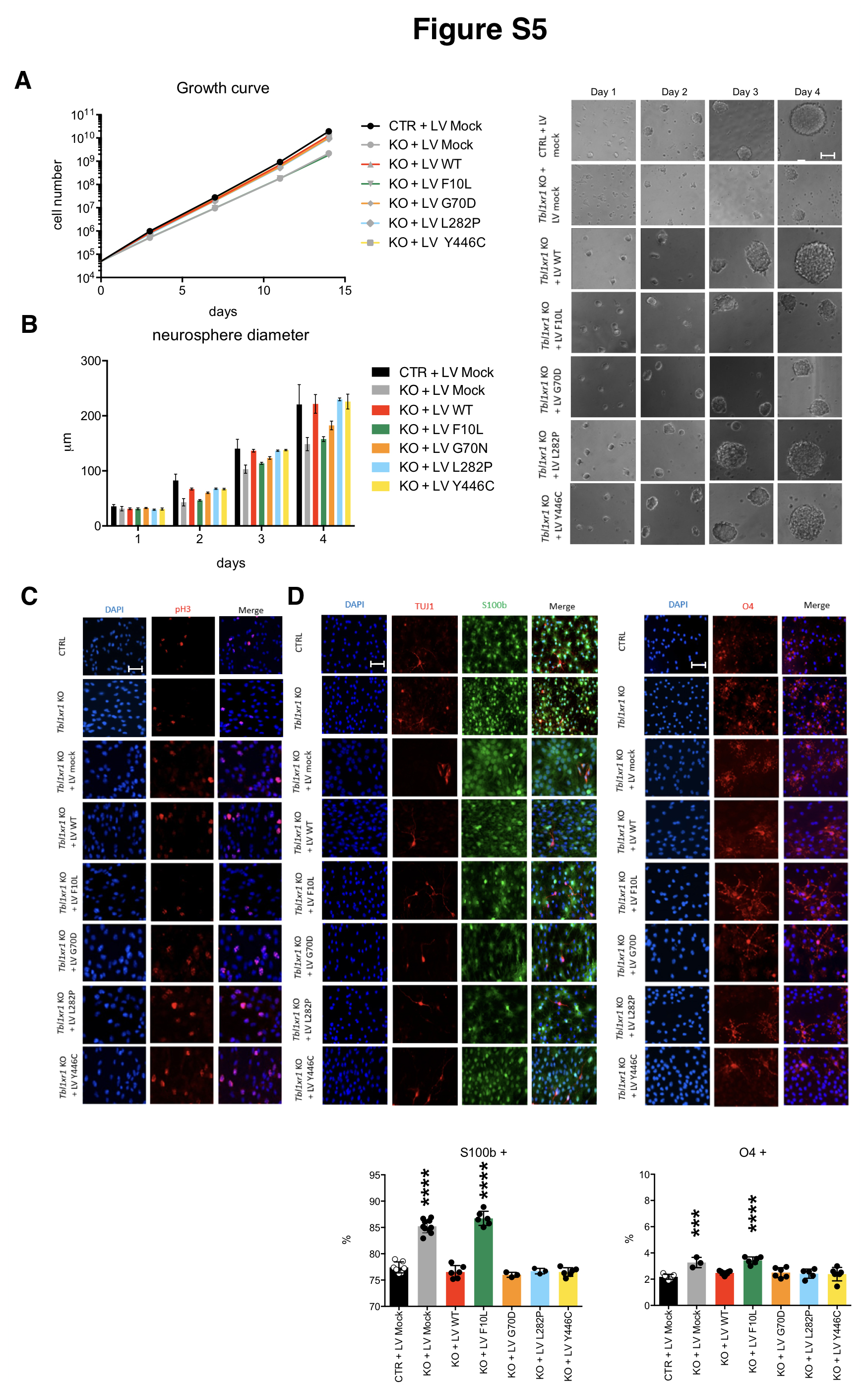

Supplement: Supplementary Figure 5 — Additional characterization of the complementation of Tbl1xr1 KO neural stem cells. (A) Growth curve of the indicated adherent in vitro NSCs, see also Figure 5B. (B) Left, histograms showing sphere’s diameters of indicated NSCs cultured as neurospheres at the indicated days in vitro after disaggregation. STATS. Right, examples of microphotographs of the neurospheres of the indicated NSCs cultured at the indicated days in vitro after disaggregation. (C) Immunocytochemistry of the indicated proliferating NSCs for phosphor histone 3 (PH3) counterstained with DAPI. Quantification is shown in Figure 5C. (D) Immunocytochemistry of the indicated proliferating NSCs for TUJ1 (red) and S100b (green) counterstained with DAPI (left) and O4 counterstained with DAPI (right). Quantification (shown as mean + s.e.m. with dots representing individual samples): TUJ1: is shown in Figure 5C; S100b: Ctrl + LV Mock vs.: KO + LV Mock ****p < 0.0001; KO + LV WT p = 0.5496; KO + LV F10L ****p < 0.0001; KO + LV G70D p = 0.2906; KO + LV L282P p = 0.8682; KO + LV Y446C p = 0.5420; one-way ANOVA with Dunnett’s multiple comparisons test. O4: Ctrl + LV Mock vs.: KO + LV Mock ∗∗∗p = 0.0002; KO + LV WT p = 0.3449; KO + LV F10L ****p < 0.0001; KO + LV G70D p = 0.3247; KO + LV L282P p = 0.6425; KO + LV Y446C p = 0.7304; one-way ANOVA with Dunnett’s multiple comparisons test. Scale bars: (A) 60 μm; (C,D) 50 μm. [file Image_5.JPEG]
